# GAME9 regulates the biosynthesis of steroidal alkaloids and upstream isoprenoids in the plant mevalonate pathway

**DOI:** 10.1038/ncomms10654

**Published:** 2016-02-15

**Authors:** Pablo D. Cárdenas, Prashant D. Sonawane, Jacob Pollier, Robin Vanden Bossche, Veena Dewangan, Efrat Weithorn, Lior Tal, Sagit Meir, Ilana Rogachev, Sergey Malitsky, Ashok P. Giri, Alain Goossens, Saul Burdman, Asaph Aharoni

**Affiliations:** 1 Department of Plant and Environmental Sciences, Weizmann Institute of Science, Rehovot 7610001, Israel; 2 Department of Plant Pathology and Microbiology, The Robert H. Smith Faculty of Agriculture, Food and Environment, The Hebrew University of Jerusalem, Rehovot 76100, Israel; 3 Department of Plant Systems Biology, Flanders Institute for Biotechnology (VIB), Gent B-9052, Belgium; 4 Department of Plant Biotechnology and Bioinformatics, Ghent University, Gent B-9052, Belgium; 5 Plant Molecular Biology Unit, Division of Biochemical Sciences, Council of Scientific and Industrial Research-National Chemical Laboratory, Pune 411008, India

## Abstract

Steroidal glycoalkaloids (SGAs) are cholesterol-derived molecules produced by solanaceous species. They contribute to pathogen defence but are toxic to humans and considered as anti-nutritional compounds. Here we show that *GLYCOALKALOID METABOLISM 9* (*GAME9*), an APETALA2/Ethylene Response Factor, related to regulators of alkaloid production in tobacco and *Catharanthus roseus*, controls SGA biosynthesis. *GAME9* knockdown and overexpression in tomato and potato alters expression of SGAs and upstream mevalonate pathway genes including the cholesterol biosynthesis gene *STEROL SIDE CHAIN REDUCTASE 2* (*SSR2*). Levels of SGAs, C24-alkylsterols and the upstream mevalonate and cholesterol pathways intermediates are modified in these plants. Δ(7)-STEROL-C5(6)-DESATURASE (C5-SD) in the hitherto unresolved cholesterol pathway is a direct target of GAME9. Transactivation and promoter-binding assays show that GAME9 exerts its activity either directly or cooperatively with the SlMYC2 transcription factor as in the case of the *C5-SD* gene promoter. Our findings provide insight into the regulation of SGA biosynthesis and means for manipulating these metabolites in crops.

## Abstract (web summary)

Steroidal glycoalkaloids (SGAs) accumulate in solanaceous plants and contribute to plant defence but are toxic to humans. Here the authors show that the GAME9 transcription factor is a regulator of the SGA biosynthetic pathways providing a potential way to manipulate SGA levels in crops.

## Graphical abstract

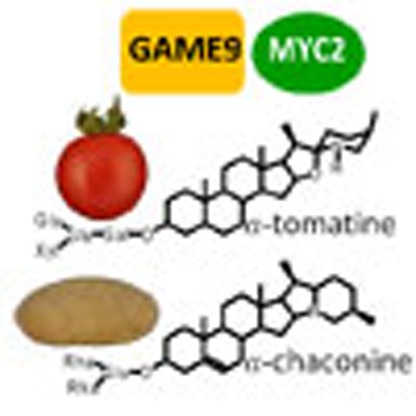

Steroidal alkaloids (SAs) and their glycosylated forms (steroidal glycoalkaloids; SGAs) are nitrogen-containing toxic compounds occurring primarily in the Solanaceae and Liliaceae plant families^1^. This class of metabolites is produced in Solanaceae vegetable crops such as potato, tomato and eggplant. Although SGAs contribute to plant resistance to a wide range of pathogens and predators, including bacteria, fungi, oomycetes, viruses, insects and animals^2^, some are considered as anti-nutritional compounds to humans due to their toxic effects^3^,^4^.

In potato, *α*-chaconine and *α*-solanine comprise >90% of the total SGA content in the tubers. Nevertheless over 50 different SAs have been identified in a variety of potato wild species and commercial cultivars^5^,^6^. In tomato, *α*-tomatine and dehydrotomatine are the major SGAs in green tissues, while esculeosides are predominant in the red ripe fruit^7^,^8^,^9^. About 100 SAs have been reported in different tissues and developmental stages of tomato^8^,^10^,^11^,^12^. Explored to a lesser extent, *α*-solasonine and *α*-solamargine are the two major SGAs found in eggplant^13^,^14^. Early studies of SGA biosynthesis in potato reported on the characterization of three glycosyltransferases (SGT1, SGT2 and SGT3) that are involved in the addition of sugar moieties on the aglycone solanidine, leading to specific synthesis of either *α*-solanine or *α*-chaconine^15^,^16^,^17^,^18^. In tomato, the first gene reported in the synthesis of SGAs was the potato *SGT1* homolog, GLYCOALAKLOID METABOLISM 1 (*GAME1*), which adds a galactose to the aglycone tomatidine^8^.

Recently, Itkin *et al.*^5^ reported a set of *GLYCOALKALOID METABOLISM* (*GAME*) genes that participate in the core pathway producing SGAs in both potato and tomato. Consequently, an elaborated pathway for SGA biosynthesis in the Solanaceae family, starting from the precursor cholesterol up to the SGAs, was proposed^5^. Extensive functional characterization suggested that cholesterol undergoes several hydroxylation, oxidation, transamination and glycosylation steps to generate SGAs. The *GAME* genes were found to be located physically close to each other in the genome and thus organized in a form of metabolic gene clusters. In tomato six *GAME* genes are positioned in a cluster on chromosome 7, whereas two other neighboring genes on chromosome 12. Furthermore, three additional genes, encoding cytochrome P450s (P450s), not belonging to these clusters, were also associated with SGA biosynthesis (*GAME7*, *GAME8a* and *GAME8b*). In potato, four SGA-related genes are located on chromosome 7 and two on chromosome 12. In tomato, the *GAME* genes include P450s [*GAME7*, *GAME8a*, *GAME8b*, *GAME6* (chromosome 7 cluster) and *GAME4* (chr. 12)], a dioxygenase (*GAME11*; chr. 7) involved in the hydroxylation and oxidation of the cholesterol skeleton and a transaminase protein (*GAME12*; chr. 12) required for the incorporation of the nitrogen atom into the SA aglycone. Finally, glycosyltransferases (*GAME1*, *GAME17*, *GAME18* and *GAME2*; chr. 7) required for generating the sugar moieties that decorate the SA aglycone were also among the clustered genes.

Cholesterol, produced through the cytosolic isoprenoid mevalonate pathway is a key precursor in the biosynthesis of SGAs. In sharp contrast to other kingdoms, the pathway leading to cholesterol biosynthesis in plants is only partially understood. Very recently, research related to SGA biosynthesis advanced our knowledge regarding the pathway to cholesterol formation in SGA-producing Solanaceae species. Sawai *et al.*^19^ demonstrated that STEROL SIDE CHAIN REDUCTASE 2 (SSR2) exhibits Δ^24(25)^ reductase activity that converts cycloartenol to cycloartanol in the first committed step towards cholesterol formation. Hence, SSR2 directs the pathway towards cholesterol and SAs instead of alkylated sterol biosynthesis^19^. On the other hand, STEROL METHYLTRANSFERASE1 (SMT1) directs the pathway towards C-24 alkylsterols by adding a methyl group at the C-24 position of the cycloartenol side chain^20^. Overexpression of a soybean *SMT1* in potato plants therefore increased the metabolic flux of cycloartenol into alkylated sterols at the expense of cholesterol^21^.

In contrast to the intense research related to structural genes of the pathway, the transcriptional regulation of SGA biosynthesis and its cholesterol precursor pathway is utterly unclear. Some transcription factors have been identified that regulate the biosynthesis of other classes of alkaloids in different plant species^22^,^23^,^24^,^25^,^26^,^27^,^28^,^29^ such as the one represented by the APETALA2/Ethylene Response Factors (AP2/ERF) family members. The AP2/ERF transcription factor ORCA3 regulates the biosynthesis of terpenoid indole alkaloids (TIAs) in *Catharanthus roseus*^22^. *ORCA3* gene expression is induced by jasmonate and is regulated by direct binding of the basic helix-loop-helix (bHLH) transcription factor CrMYC2 to the *ORCA3* gene promoter^30^. Close homologs of ORCA3 in *Nicotiana tabacum* present in the *NIC2* locus were associated with nicotine levels in the tobacco leaf and have been used extensively in breeding of low-nicotine tobacco lines^31^. Specifically, the *NIC2* locus comprises at least seven ERF transcription factors that regulate the expression of structural genes in the biosynthesis of nicotine. In the *nic2* mutant, this *ERF* gene cluster is deleted, resulting in a low-nicotine phenotype^24^. Genes present in the *NIC2* locus include *ERF189* and *ERF221* (also known as ORC1 (ref. 23)). Overexpression of *ERF189* and *ERF221/ORC1* was sufficient to stimulate nicotine biosynthesis in tobacco plants^24^,^26^. Members of the ERF family of transcription factors can recognize different GC-rich boxes in the promoters of target genes activating their transcription^32^,^33^.

In this study, we identified *GLYCOALKALOID METABOLISM 9* (*GAME9*), an AP2/ERF transcription factor that regulates the biosynthesis of steroidal alkaloids in Solanaceae plants. We found that *GAME9* is part of an *ERF*-gene cluster existing in potato and tomato. Transactivation and promoter binding assays as well as transgenic tomato and potato plants revealed that GAME9 controls SGA biosynthesis as well as several upstream mevalonate and cholesterol precursor pathway genes. Furthermore, GAME9 exerts its activity either directly or through co-binding with the SlMYC2 transcription factor to promoters of downstream target genes. These findings provide insight into the transcriptional regulation of SGAs in Solanaceae plants as well as a base for engineering these anti-nutritional compounds in plants.

## Results

### Initial evidence of *GAME9* association with SGA biosynthesis

**Figure 1:**
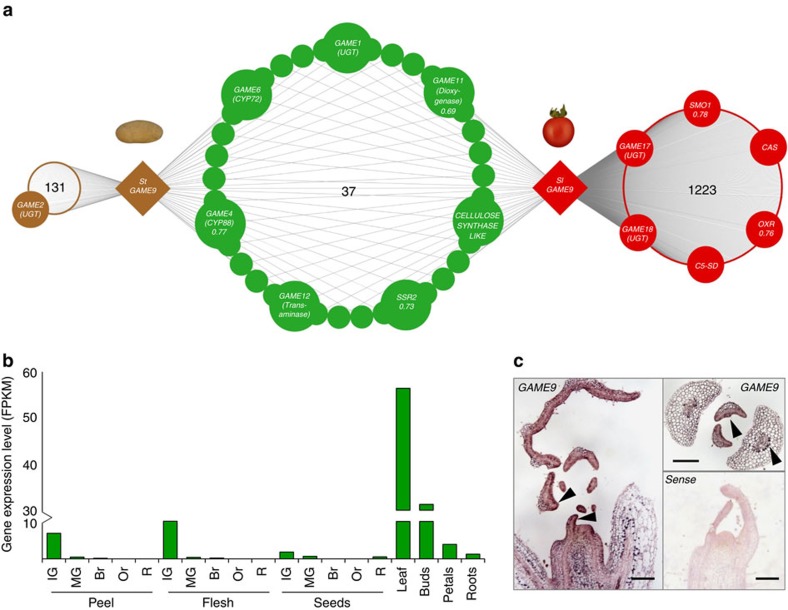
Co-expression network of *GAME9* in potato and tomato. (**a**) Using RNA-Seq transcriptome data from potato and tomato, we found that *GAME9* was co-expressed with most of the SGA biosynthetic genes. A total of 1,260 and 168 genes were co-expressed with *GAME9* in tomato and potato, respectively. Out of them, thirty seven were shared homologs co-expressed in both potato and tomato (see Supplementary Data 1 and Supplementary Table 1). *C5-SD*: *Δ(7)-STEROL-C5(6)-DESATURASE*, *SMO1*: *METHYLSTEROL MONOOXYGENASE 2-2-LIKE*, *OXR*: 3-*β HYDROXYSTEROID DEHYDROGENASE*, *CAS*: *CYCLOARTENOL SYNTHASE*. (**b**) Normalized expression profile (from RNA-sequencing) of *GAME9* in different tomato tissue types and developmental stages. Br, breaker; IG, immature green; MG, mature green; Or, orange; R, ripe. FPKM: Fragments Per Kilobase of transcript per Million mapped reads. (**c**) *In situ* mRNA hybridization showing *GAME9* expression in wild-type tomato shoot meristems. Both longitudinal and transverse sections are shown. Sense *GAME9* probe is shown as a control. Arrows indicate higher expression in the vascular system and the outer layer of the leaf blade. Scale bars: 200 μm.

In a previous study, we discovered an AP2/ERF type transcription factor displaying a similar expression pattern to the *GAME1* and *GAME4* genes of the tomato and potato SGA biosynthetic pathway (both *GAME* genes used as baits in co-expression analysis)^5^. To examine the possible association of this regulator (termed GLYCOALKALOID METABOLISM 9; GAME9) in the control of SGA biosynthesis, we carried out combined co-expression analysis using potato and tomato transcriptome data (see Methods for details on co-expression analysis). A total of 1,260 and 168 genes were co-expressed with *GAME9* in tomato (*SlGAME9*) and potato (*StGAME9*), respectively (Fig. 1; Supplementary Data 1). Thirty seven homologous genes were co-expressed with *GAME9* in both potato and tomato (Fig. 1a; Supplementary Table 1). Among the co-expressed genes, we found all those previously associated with SGA biosynthesis in potato (i.e., *GAME2*, *GAME11*, *GAME6*, *GAME1*, *GAME12* and *GAME4*) and tomato (*GAME11*, *GAME6*, *GAME17*, *GAME1*, *GAME18*, *GAME12* and *GAME4*) (Fig. 1a). Genes encoding HMGR and SQS^34^,^35^, involved in the synthesis of isoprenoid precursors in the mevalonate pathway, were not co-expressed with *GAME9* in either species (Supplementary Data 1). Interestingly, the phytosterols and cholesterol biosynthesis related genes *CYCLOARTENOL SYNTHASE* (*CAS*) and *SSR2* were co-expressed with *GAME9* in tomato (*r*-value≥0.73) while only *SSR2* was co-expressed with the potato *GAME9* gene (Supplementary Data 1). When examined across 19 different tomato tissue types, *SlGAME9* was highly expressed in leaf and flower buds. In fruit tissues, it was expressed early, predominantly in the immature stages of development (Fig. 1b) while displaying some, albeit relatively low level of expression in petals and root tissues. The expression pattern of *SlGAME9* was analyzed using RNA *in situ* hybridization. In 13-day-old tomato shoots, *SlGAME9* was expressed in both young leaves and throughout the vascular system. *SlGAME9* expression was also detected in mature leaves, mostly in the outer epidermis layer of the blade (Fig. 1c).

### *GAME9* lies within a QTL previously linked to SGA content

**Figure 2:**
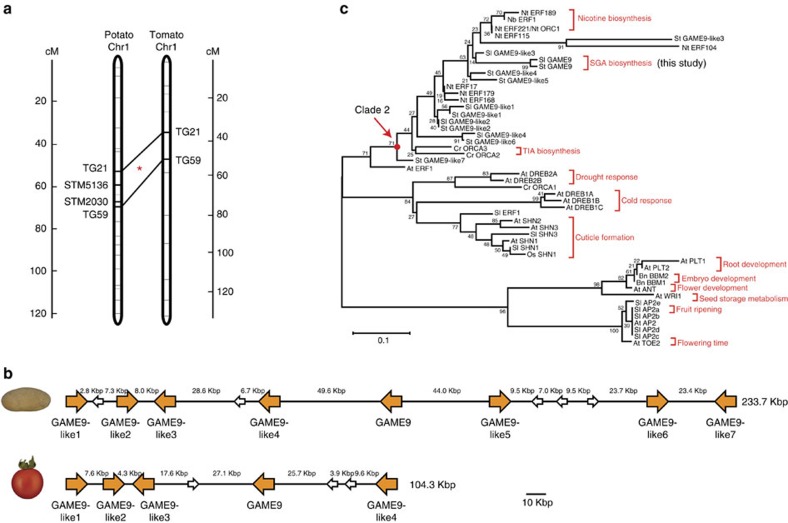
*GAME9* is part of an *ERF*-gene cluster in the Solanaceae and is related to other alkaloid-associated regulatory genes. (**a**) A major QTL involved in the synthesis of SGAs is present on potato chromosome 1. The QTL is flanked by the SSR markers STM5136 and STM2030^36^. Using a comparative map viewer we identified the markers TG21 (ST4.03ch01:62729267..62729866) and TG59 (ST4.03ch01:71035810..71036389) and the corresponding region in tomato [markers TG21 (SL2.50ch01:78511596..78512173) and TG59 (SL2.50ch01:85194698..85195223)]. *GAME9 was found to be located in this QTL region in both potato and tomato. (**b**) Schematic presentation of *GAME9* and *GAME9*-like genes in chromosomal regions of potato and tomato. In these regions, we found clusters of *ERF* genes spanning a ∼230 kbp region (ST4.03ch01:69392381..69626333) in potato and a region of ∼104 kbp in tomato (SL2.50ch01:83966837..84071427). (**c**) Phylogenetic analysis of GAME9 and other related AP2-family proteins from tomato (Sl), potato (St), tobacco (Nt), *N. benthamiana* (Nb), periwinkle (Cr), rice (Os), *Brassica napus* (Bn), and *Arabidopsis* (At). The evolutionary history was inferred using the neighbor-joining method in MEGA6 (ref. 58). The percentage of replicate trees in which the associated taxa clustered together in the bootstrap test (1000 replicates) is shown next to the branches. Accession numbers can be found in Supplementary Table 8.

Identification of QTLs linked to total SGA content in potato tubers has been of high interest in breeding of new potato cultivars. Sørensen *et al.*^36^ reported a highly significant QTL on chromosome 1 that explained a major proportion of the SGA content in potato tubers (both in dark and light exposed tubers). Considering that *GAME9* is located on chromosome 1 (Solyc01g090340 and PGSC0003DMG400025989, in tomato and potato, respectively), we suspected that it might be associated with this earlier reported QTL region. The potato QTL was flanked by the simple sequence repeat (SSR) markers STM5136 and STM2030 (ref. 36). Using these markers and the Comparative Map Viewer and Genome Browser tools available in The Sol Genomics Network (SGN, http://solgenomics.net), we identified the corresponding chromosomal region spanning 6.6 Mbp on chromosome 1 of tomato [between markers TG21 and TG59 (Fig. 2a)]. In both species, *GAME9* was located inside these QTL regions, and moreover, as part of a cluster of AP2/ERF transcription factors. In potato, a cluster spanning ∼230 kilobase pair (kbp) genomic region includes *GAME9* together with seven *GAME9*-like transcription factors, whereas in tomato, a region of ∼104 kbp contains *GAME9* and additionally four *GAME9*-like genes (Fig. 2b).

Phylogenetic analysis showed that GAME9 and GAME9-like proteins are part of the ERF IXa subfamily^37^ divided earlier by Shoji *et al.*^24^ into two separate clades. GAME9 and the GAME9-like proteins are part of clade 2 that includes the tobacco *NIC2* locus protein ERF189 involved in the synthesis of the pyridine alkaloid nicotine. The same clade also includes ORCA3 and ORCA2, both transcription factors involved in the synthesis of TIAs in *C. roseus*^22^,^38^ (Fig. 2c). Two other members of the ERF IXa subfamily clade 2 are also involved in the control of nicotine biosynthesis, namely, the tobacco ERF221 (ORC1)^23^ and the *Nicotiana benthamiana* ERF1^25^. Thus, *GAME9* represents a potential third case in which proteins of this clade control the biosynthesis of different classes of alkaloids.

### Altering *GAME9* expression impacts the levels of major SGAs

**Figure 3:**
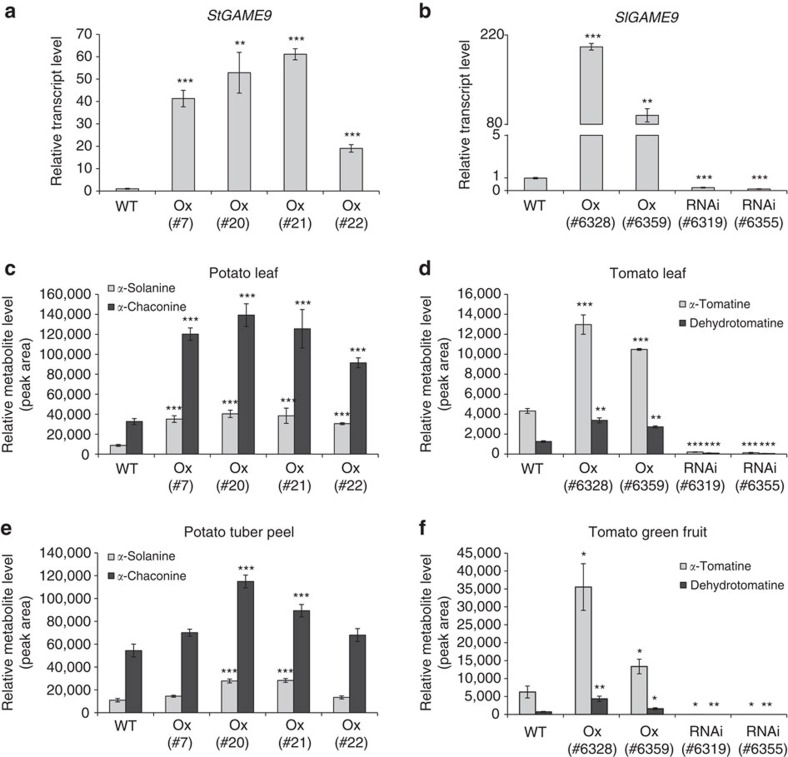
Changes in *GAME9* expression in potato and tomato results in altered levels of the predominant SGAs. (**a**,**b**) *GAME9* gene expression (qRT-PCR) in *GAME9*-Ox (overexpression) and *GAME9*-RNAi (silencing) lines in potato (**a**) and tomato (**b**) leaf tissue. WT: wild-type. Potato *GAME9*-Ox independent lines (#7, #20, #21 and #22). Tomato *GAME9*-Ox independent lines (#6328 and #6359) and *GAME9*-RNAi (#6319 and #6355). (**c**–**f**) Levels of *α*-solanine and *α*-chaconine in leaves (**c**) and peel (**e**) of potato tubers of *GAME9*-Ox lines and levels of *α*-tomatine and dehydrotomatine in leaves (**d**) and green fruit (**f**) of *GAME9*-RNAi and *GAME9*-Ox tomato lines, determined by LC-MS. Values represent means±s.e. (*n*=3). Student's *t*-test was used to assess whether the transgenic lines significantly differ from wild-type plants: *P value<0.05; **P value<0.01; ***P value<0.001.

To provide additional evidence regarding the role of *GAME9* in SGA biosynthesis, we generated transgenic tomato lines in which *GAME9* was silenced (*GAME9*-RNAi) or overexpressed (*GAME9*-Ox). Transgenic potato lines overexpressing *GAME9* were also generated. Real-Time PCR analysis in leaves showed *GAME9* expression was significantly higher in *GAME9*-Ox lines from potato and tomato, and was decreased in the *GAME9*-RNAi tomato lines (Fig. 3a,b). SGAs profiling was carried out on extracts of tomato and potato leaves, skin of potato tubers and tomato fruits by Liquid Chromatography Mass Spectrometry (LC-MS). In leaves of potato *GAME9*-Ox lines, the levels of *α*-solanine and *α*-chaconine increased between 3.5–4.6 fold and 2.8–4.2 fold, respectively as compared to leaves of wild-type plants (Fig. 3c). Likewise, in tuber skin isolated from the same potato lines, we detected an increase in *α*-solanine levels (up to 1.2–2.6 fold) and *α*-chaconine (up to 1.2–2.1 fold) (Fig. 3e). In tomato leaves, the levels of *α*-tomatine and dehydrotomatine were significantly increased (2.4–3 fold and 2.1–2.7 fold, respectively) in *GAME9*-Ox lines, whereas in *GAME9*-RNAi lines there was a reduction in the levels of *α*-tomatine (21–32 fold) and dehydrotomatine (13–21 fold) as compared to wild-type plants (Fig. 3d). Similarly, in green fruit from the same tomato lines, there was an increase in *α*-tomatine (2.1–5.7 fold) and dehydrotomatine (2.2–6.1 fold) in *GAME9*-Ox lines and a reduction in *α*-tomatine (45–47 fold) and dehydrotomatine (37–50 fold) in *GAME9*-RNAi lines compared to wild-type tomato plants (Fig. 3f).

### Impact on the mevalonate pathway and its branches

**Figure 4:**
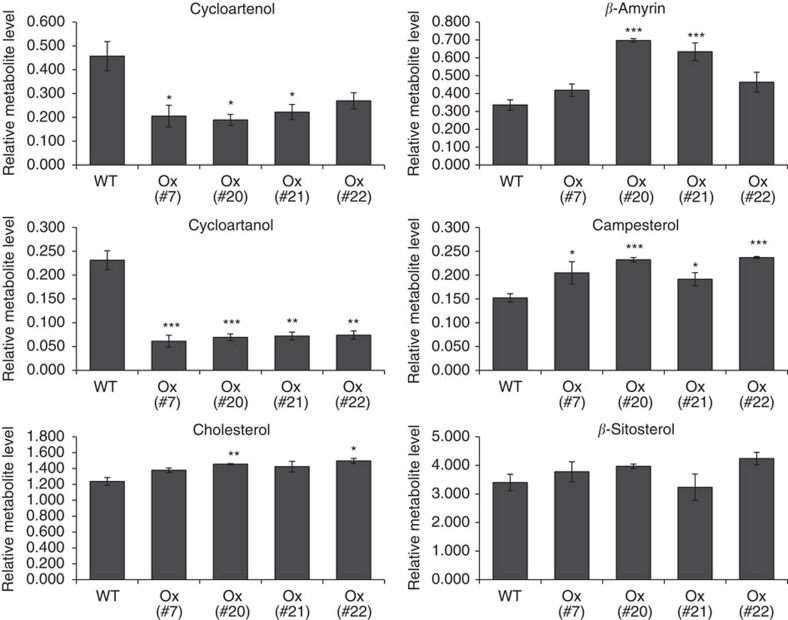
Cholesterol and other sterols levels in potato *GAME9*-Ox lines. Relative abundance of six sterols in leaves of four independent *GAME9*-Ox plant lines as compared to wild-type (WT) measured using GC–MS. Epicholesterol was used as an internal standard. Relative metabolite levels are expressed as ratios of peak areas compared to internal standard. Values represent means±s.e. (*n*=3). Student's *t*-test was used to assess whether the transgenic lines significantly differ from wild-type plants: *P value<0.05; **P value<0.01.

We envisaged that regulation of SGA content by GAME9 is achieved, at least partially, by regulating the flux through the mevalonate pathway and its branches. These include C-24 alkylated phytosterols (e.g., campesterol and *β*-sitosterol), non-alkylated sterols (primarily cholesterol, which is the precursor for SGA biosynthesis), and the triterpenoid branch. Gas Chromatography Mass Spectrometry (GC–MS) was employed to profile the various metabolic intermediates in leaves of the four potato *GAME9-*Ox lines. Overexpression of *GAME9* in potato resulted in a significant decrease in levels of cycloartenol and cycloartanol, early intermediates in cholesterol biosynthesis (Fig. 4). Cholesterol itself showed a slight, but significant increase in leaves of the *GAME9-*Ox lines (Fig. 4). Interestingly, *β*-amyrin and campesterol contents were also increased, yet, *β*-sitosterol was detected in levels similar to those in leaves of wild-type plants (Fig. 4). These observations point to increased flux to cholesterol, the triterpene *β*-amyrin as well as to a certain part of phytosterol biosynthesis (i.e., campesterol) due to *GAME9* overexpression.

**Figure 5:**
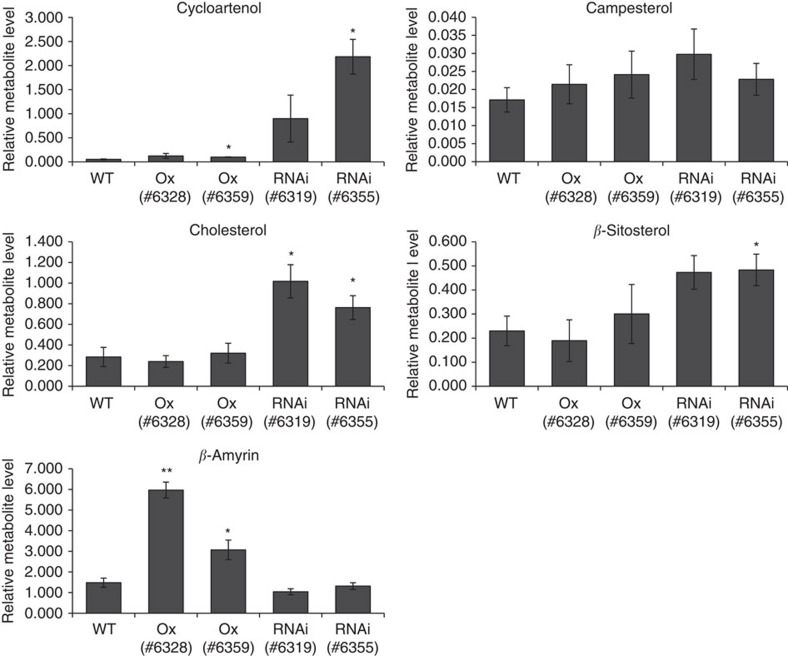
Cholesterol and other sterols levels in tomato *GAME9*-Ox and *GAME9*-RNAi lines. Relative abundance of five sterols in leaves of two independent *GAME9*-Ox and two independent *GAME9*-RNAi plant lines as compared to wild-type (WT) measured using GC–MS. Epicholesterol was used as an internal standard. Relative metabolite levels are expressed as ratios of peak areas compared to internal standard. Values represent means±s.e. (*n*=3). Student's *t*-test was used to assess whether the transgenic lines significantly differ from wild-type plants: *P value<0.05; **P value<0.01.

Similarly, in tomato we detected altered sterol composition when *GAME9* was either overexpressed or downregulated (Fig. 5). For instance, in *GAME9*-Ox lines there was a significant increase in *β*-amyrin level. On the other hand, *GAME9*-RNAi lines had an increase in cycloartenol, cholesterol and *β*-sitosterol content (Fig. 5). Campesterol did not show any significant differences in *GAME9*-altered plants compared to wild-type.

### Gene expression analysis in plants misexpressing *GAME9*

**Figure 6:**
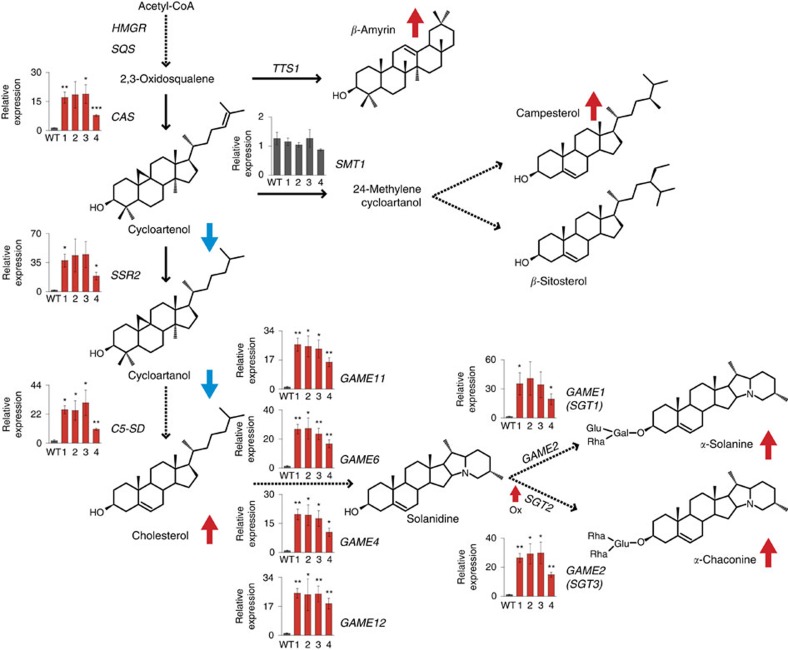
Expression of genes involved in the synthesis of SGAs and sterol precursors in potato leaves derived from *GAME9*-Ox lines determined by qRT-PCR and RNA-Seq analyses. Schematic view of the sterol and SGA biosynthetic pathway. Dashed arrows represent multiple biosynthetic reactions whereas solid arrows represent a single step. The graphs next to each gene name show expression levels in wild-type (WT) and four *GAME9*-Ox independent lines (lines 1:#7, 2:#20, 3:#21 and 4:#22) determined by qRT-PCR. For genes where qRT-PCR data is not presented, it is due to technical reasons and differential expression awaits confirmation. According to RNA-Seq data, expression of *HMGR*, *SQS*, *TTS1* was similar, and *SGT2* was overexpressed in *GAME9*-Ox lines. Arrows next to each compound represent an increase (red) or decrease (blue) in potato *GAME9*-Ox lines (see Figs 3 and 4). Values represent means±s.e. (*n*=3). Student's *t*-test was used to assess whether the transgenic lines significantly differ from wild-type plants: *P value<0.05; **P value<0.01; ***P value<0.001.

We used quantitative Real-Time PCR (qRT-PCR) to examine the expression level of SGA biosynthetic genes and those in the mevalonate and downstream pathways (towards triterpenoids, phytosterol and cholesterol biosynthesis) in the *GAME9* altered plants. In potato, *GAME9* overexpression did not change the expression of genes involved in the upper mevalonate pathway (i.e., *HMGR*, *SQS*)^34^,^35^, *β*-amyrin (the potato homolog of *TRITERPENOID SYNTHASE 1*, *TTS1* (ref. 39)) and campesterol/*β*-sitosterol (*SMT1*) (Fig. 6; Supplementary Table 2). However, genes acting downstream to 2,3-oxidosqualene, towards the formation of sterols, including *CAS*, *SSR2* and *C5-SD*, were upregulated in the *GAME9*-Ox potato lines. Similarly, in the same lines, the *GAME* genes responsible for the synthesis of the solanidine aglycone (*GAME11*, *GAME6*, *GAME4* and *GAME12*) and the subsequent glycosylation (*GAME1*, *GAME2* and *SGT2*) were all significantly upregulated (Fig. 6; Supplementary Table 2).

**Figure 7:**
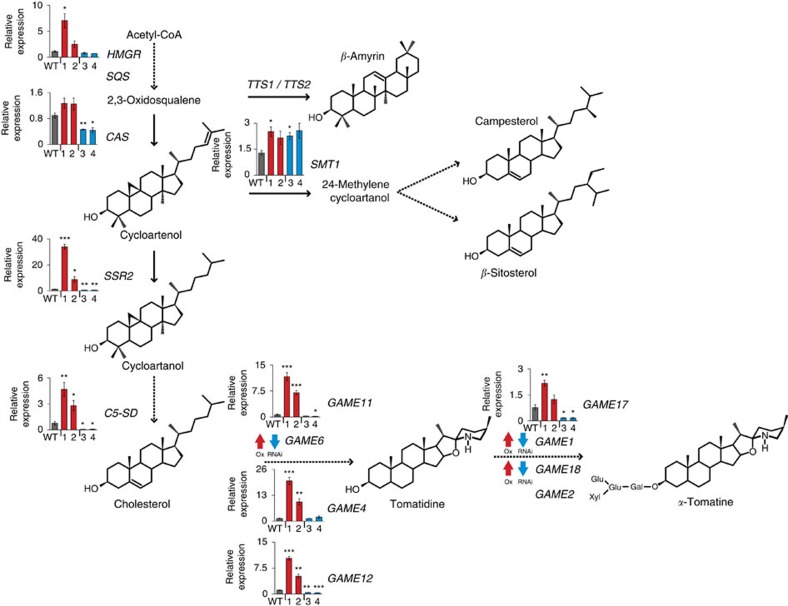
Expression of genes involved in the synthesis of SGAs and sterol precursors in tomato leaves derived from *GAME9*-Ox and *GAME9*-RNAi lines. A schematic view of the sterol and SGA biosynthetic pathways. Dashed arrows represent multiple biosynthetic reactions whereas solid arrows represent a single step. Graphs next to each gene name show expression levels in wild-type (WT), two *GAME9*-Ox (lines 1:#6328 and 2:#6359) and two *GAME9*-RNAi lines (line 3:#6319 and 4:#6355) determined by qRT-PCR. For genes where qRT-PCR data is not presented, it is due to technical reasons and differential expression awaits confirmation. According to RNA-Seq data, expression of *SQS*, *TTS1*, *TTS2* and *GAME2* was similar in *GAME9*-Ox and *GAME9*-RNAi tomato lines. For other genes, expression is represented based on RNA-Seq data, with arrows next to each gene depicting an increase (red) or decrease (blue) in *GAME9*-Ox and *GAME9*-RNAi lines, respectively. Values represent means±s.e. (*n*=3). Student's *t*-test was used to assess whether the transgenic lines significantly differ from wild-type plants: *P value<0.05; **P value<0.01; ***P value<0.001.

In tomato, we found that when *GAME9* was either silenced or overexpressed, expression of *HMGR*, *CAS*, *SSR2* and *C5-SD* was significantly altered. However, expression of *TTS1* and *TTS2* (ref. 39) involved in the triterpene *β*-amyrin formation was not affected. Altered expression of *GAME9* did not affect *SQS* but upregulated *SMT1* expression (Fig. 7; Supplementary Table 3). Finally, 7 out of 8 examined *GAME* genes involved in the synthesis of the SGA aglycone tomatidine and its glycosylation were altered in expression, at all times correlating with the *GAME9* transcript levels in tomato leaf tissues (Fig. 7).

### Transcriptome changes in *GAME9*-Ox and *GAME9*-RNAi lines

To obtain a more global picture of genes that are downstream of GAME9 and to understand more precisely the metabolic pathways under its control, we performed RNA-sequencing (RNA-Seq) in leaf tissue of *GAME9*-RNAi and *GAME9*-Ox tomato lines and wild-type. Transcriptome analysis was also conducted on leaves of potato lines overexpressing *GAME9* and wild-type ones. Silencing of *GAME9* in tomato resulted in 931 genes that were downregulated [fold change log_2_ (RNAi/WT)<−0.5; Supplementary Data 2]. When *GAME9* was overexpressed, 1,002 genes were upregulated in tomato [fold change log_2_ (Ox/WT)>0.5]. *GAME9* overexpression in potato, led to upregulation of 1,829 genes [fold change log_2_ (Ox/WT) >0.5; Supplementary Data 2].

A concise set of 27 genes (including *GAME9*) was found in common between the down- and upregulated genes in the *GAME9*-RNAi and *GAME9*-Ox tomato lines, respectively (Supplementary Table 4). Among these, we found a significant representation of SGA biosynthetic genes (*GAME*s), explicitly those located in the metabolic gene cluster in tomato chromosome 7 (*GAME*s *1*, *6*, *11*, *17* and *18*; Supplementary Fig. 1). This gene set also contained an additional gene in the SGAs cluster on chromosome 7, a sequence with homology to cellulose synthase family proteins (Solyc07g043390). The *CELLULOSE SYNTHASE LIKE* transcript was also found to be significantly co-expressed with *GAME9* in both tomato and potato (Fig. 1; Supplementary Table 1). Four genes out of the 27 could be associated with sterol metabolism, possibly phytosterol or cholesterol biosynthesis^20^ (Supplementary Table 4). Recent work reported one of the four genes, namely *SSR2*, a sterol side chain reductase catalyzing the first committed step towards cholesterol formation in the Solanaceae^19^ (the conversion of cycloartenol to cycloartanol; Fig. 6). The three additional genes include homologs of a *Δ(7)-STEROL-C5(6)-DESATURASE* (*C5-SD*), *METHYLSTEROL MONOOXYGENASE 2-2-LIKE* (*SMO*1) and a *3-β-HYDROXYSTEROID DEHYDROGENASE* (*OXR*) (Supplementary Table 4). Out of these 4 sterol metabolism associated genes, *SSR2* was co-expressed with *GAME9* in both potato and tomato, while the other three (i.e., *C5-SD*, *SMO1* and *OXR*) were significantly co-expressed with the *GAME9* transcript in tomato (Fig. 1). Finally, among the 27 genes set we found a homolog of the *E3 UBIQUITIN-PROTEIN LIGASE RMA1H1-LIKE*. Apart from being significantly co-expressed with *GAME9* (Supplementary Data 1), this gene is related to an ERAD-type RING membrane-anchor E3 ubiquitin ligase reported to control the activity of 3-hydroxy-3-methylglutaryl-CoA reductase (HMGR)^40^, the rate-limiting enzyme in the mevalonate pathway leading to cholesterol and subsequently SGAs formation.

A set of 466 genes was found in common between the upregulated genes in the *GAME9*-Ox tomato and potato lines (Supplementary Data 2). Among them, we found represented *GAME* genes located on chromosome 7 both in potato and tomato (*GAME*s *1*, *6* and *11* and *CELLULOSE SYNTHASE LIKE*). The *SSR2, C5-SD*, *SMO1* and the *E3 UBIQUITIN-PROTEIN LIGASE RMA1H1-LIKE* were also among the genes upregulated in both the potato and tomato overexpression lines (Supplementary Data 2).

### GAME9 and SlMYC2 act synergistically in gene transactivation

**Figure 8:**
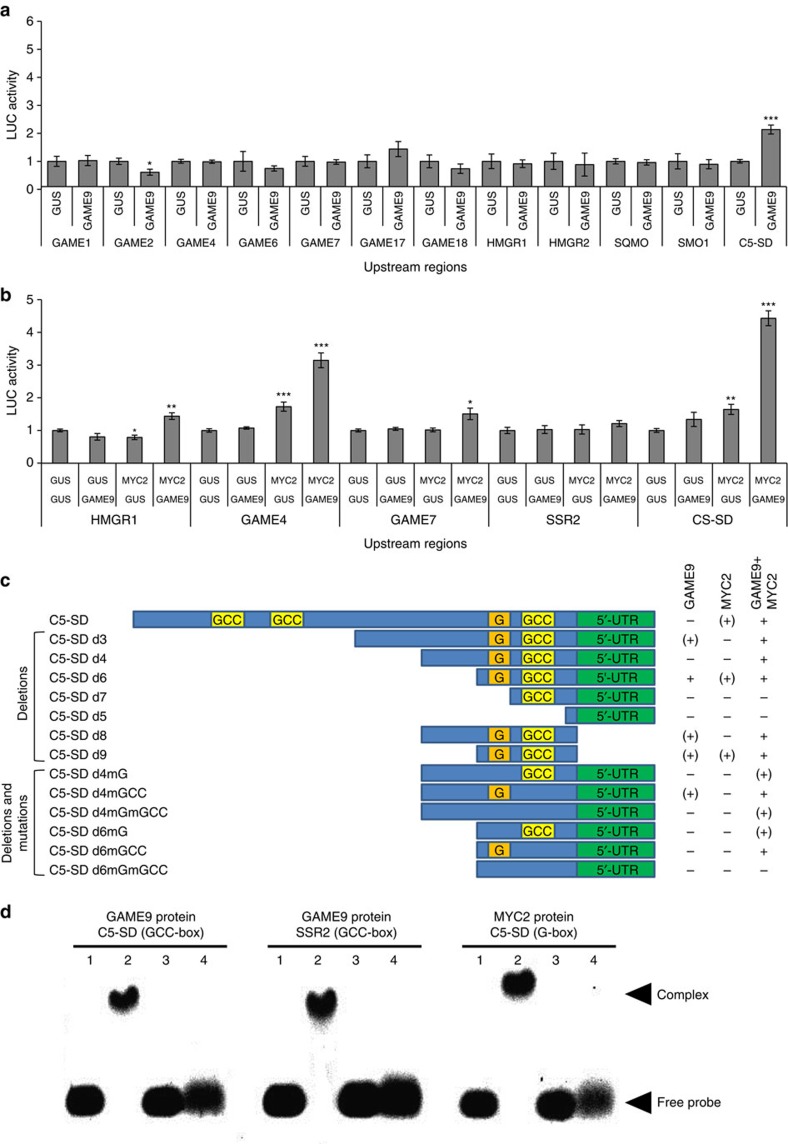
Transactivation assays of putative downstream gene promoters by the GAME9 and SlMYC2 transcription factors. (**a**) The capacity of GAME9 to transactivate 12 different promoters of candidate downstream genes was evaluated in protoplasts prepared from tobacco Bright Yellow-2 (BY-2) cells (*n*=4). A validation experiment (*n*=8) was performed, confirming the transactivation of the *C5-SD* promoter (Supplementary Fig. 2). Values in the y-axis are normalized fold-changes relative to protoplasts co-transfected with the reporter constructs and a pCaMV35S:GUS (GUS) control plasmid. For the normalization procedure, see Materials and Methods. Details for each promoter are provided in Supplementary Table 5. Student's *t*-test was used to assess whether the transgenic lines significantly differ from wild-type plants: *P value<0.05; ***P value<0.001. *GAME1*: UDP-galactosyltransferase; *GAME2*: UDP-xylosyltransferase; *GAME4*: CYP88D; *GAME6*: CYP72A; *GAME7*: CYP72A; *GAME17*: UDP-glucosyltransferase; *GAME18*: UDP-glucosyltransferase; *HMGR1*: HMG CoA reductase 1; *HMGR2*: HMG CoA reductase 2; *SQMO*: Squalene monooxygenase; *SMO1*: Methylsterol monooxygenase 2-2-like; *C5-SD*: Δ(7)-sterol-C5(6)-desaturase; *GAME9*: Ethylene responsive transcription factor. (**b**) Transactivation of SGA-related gene promoters with GAME9, SlMYC2 and the combination of both. (**c**) Transactivation of *C5-SD* promoter with deletions (d) and/or mutations (m). The -, (+) and+symbols point to activation of the promoter by GAME9/SlMYC2:—no activation (LUC activity below 1.5); (+) limited activation (LUC between 1.5 and 2-fold);+strong promoter activation (LUC activity larger than 2-fold). (**d**) *In vitro* binding of the GAME9 and SlMYC2 proteins to the G- and GCC-boxes located in the promoters of the *C5-SD* and *SSR2* genes. Electrophoretic mobility shift assays (EMSA) were performed with probes containing a GCC- or a G-box were separated on a 1% agarose gel without additional treatment (lane 1) or with 200 ng of the purified protein (lane 2). Controls: a sequence containing a mutated version of the box was loaded (lane 3) and incubated with protein (lane 4).

To study the GAME9 transactivation capacity of putative target genes upstream regions, we performed transient luciferase expression assays in tobacco protoplasts. Altogether, we assayed a total of 12 different putative promoter regions (ranging in size from 1200 to 2700, bp) of known tomato SGA genes and those involved in the mevalonate and cholesterol precursor pathways (Supplementary Table 5). In this assay GAME9 did not transactivate the putative promoter regions of any of the core SGA pathway GAME genes acting in between cholesterol and *α*-tomatine (Fig. 8a). Nevertheless, transactivation was clearly detected for the promoter of the gene *C5-SD*, putatively involved in the synthesis of cholesterol (Fig. 8a; Supplementary Fig. 2). These experiments indicated that GAME9 likely requires additional factors to control SGA production.

In tobacco, both MYC2 and ERF transcription factors are involved in the regulation of nicotine biosynthesis genes^24^,^26^,^41^,^42^. MYC2 was shown to directly bind G-box sequences in the promoters of several nicotine biosynthesis genes and to activate these genes additively with ERF189 (ref. 41). In *Catharanthus roseus*, CrMYC2 was shown to regulate TIA biosynthesis by directly binding to the *ORCA3* promoter^30^. Since G-box or G-box-like motifs could be detected in the putative promoters of several of the GAME and putative cholesterol genes, we investigated the potential additive role of SlMYC2 in the regulation of SGA biosynthesis. To this end, we cloned the tomato MYC2 (Solyc08g076930) homolog and performed additional transfection assays in tobacco protoplasts, in which SlMYC2 and GAME9 were combined to assess transactivation of a subset of five SGA biosynthesis gene promoters containing or lacking G- and/or GCC-box sequences, required for the binding of the SlMYC2 and GAME9 proteins, respectively (Supplementary Figs 3 and 4).

These assays demonstrated that SlMYC2 alone was capable to transactivate the *C5-SD* gene promoter (*ProC5-SD*) (Fig. 8b). More importantly however, a synergistic effect was observed when GAME9 was combined with SlMYC2, observed with the promoters of *C5-SD*, *GAME4*, *GAME7* and *HMGR1* pointing to a cooperative action of these two transcription factors in the regulation of SGA biosynthesis. This was further supported by the observation that SlMYC2 alone, in contrast to GAME9, could also mildly transactivate *ProGAME4*, and that with the combination of the two transcription factors, again a synergistic transactivation of *ProGAME4* was achieved (Fig. 8b). The promoters of *HMGR1* and *GAME7* were not transactivated by GAME9 or SlMYC2 alone, whereas a significant but slight (less than 1.5-fold) synergistic transactivation effect of the combination of GAME9 and SlMYC2 could be observed (Fig. 8b). The promoter of *SSR2* was not transactivated by either transcription factor alone or the combination thereof (Fig. 8b).

Analysis of the 1,550 bp promoter sequence of *C5-SD* revealed the presence of a G-box and three GCC-rich motifs (Fig. 8c, Supplementary Fig. 3). To determine if these boxes are important for the transactivation of *ProC5-SD*, we generated a series of promoter deletion constructs and assessed their transactivation by GAME9 and/or SlMYC2 in tobacco protoplasts (Fig. 8c, Supplementary Fig. 3 and Supplementary Table 6). Thereby we could pinpoint a 97-nt promoter region (C5-SD d9) sufficient for transactivation by GAME9 and SlMYC2 that contains both the G-box and a putative GCC-box. To further substantiate the importance of the G- and GCC-boxes, we created *ProC5-SD* constructs in which either one or both boxes were mutated. As expected, mutation of the 6-bp CACGTG motif of the G-box into ATGTGA was sufficient to impede promoter transactivation (Fig. 8c). Unexpectedly however, mutation of the 10-bp AGCCTGCCAC motif of the putative GCC-box into GATTACAGTC did not interfere with transactivation by GAME9 and SlMYC2. This observation indirectly correlates with the apparent absence of a GCC-motif in the *ProGAME4* sequence (Supplementary Fig. 4), which could nonetheless be transactivated by combining SlMYC2 and GAME9.

Electrophoretic mobility shift assays (EMSA) were further performed to determine the *in vitro* binding of GAME9 to its putative binding sites in *C5-SD* and *SSR2* promoters. We tested the GCC-boxes found in both promoters and compared to the binding of probes where these boxes were mutated (Fig. 8d, Supplementary Table 7). When incubated with the GAME9 protein, probes derived from both promoters (containing GCC-boxes) showed retarded bands, suggesting the formation of GAME9—DNA complexes (Fig. 8d). Furthermore, SlMYC2 protein bound to the G-box present in the promoter of *C5-SD*. When this G-box was mutated the binding was impaired (Fig. 8d, Supplementary Table 7).

### Characterization of the *C5-STEROL DESATURASE*

**Figure 9:**
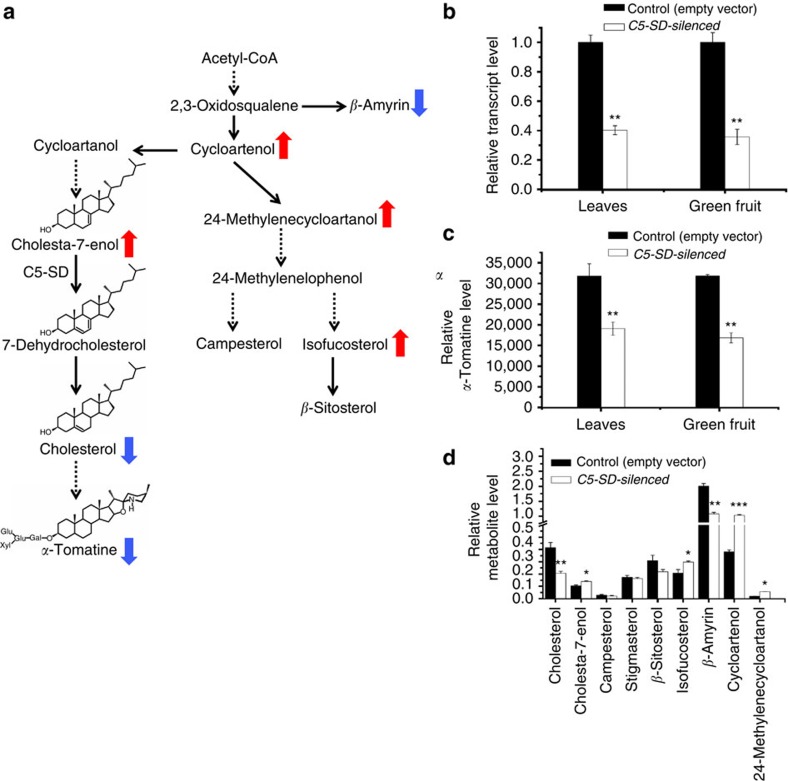
*C5-SD* silenced tomato leaves and fruit exhibit reduced cholesterol and *α-*tomatine levels. (**a**) *C5-SD* was silenced by virus induced gene silencing. Changes in sterols content for silenced *C5-SD* gene are shown with arrows next to each compound name (red: increase, blue: decrease). Dashed arrows represent multiple biosynthetic reactions whereas solid arrows represent a single step. (**b**) Relative expression level of *C5-SD* gene in VIGS-silenced plants compared to control (Empty vector harboring DEL/ROS sequences). (**c**) Level of *α*-tomatine in leaves and green fruits of *C5-SD*-silenced plants compared to control. (**d**) Relative abundance of cholesterol and other sterols levels in leaves of silenced *C5-SD* tomato as compared to control plants measured using GC–MS. Relative metabolite levels are expressed as ratios of peak areas compared to internal standard (epicholesterol). Due to very low level presence of 24-methylenecycloartanol, relative level are represented by factor 5 to basal level. Values represent mean±s.e. (*n*=3). Student's *t*-test indicates significant changes from control plants: *P value<0.05; **P value<0.01; ***P value<0.001.

Virus induced gene silencing (VIGS) was subsequently employed for functional characterization of one of the four candidate cholesterol biosynthesis genes (*C5-SD*). Real-Time PCR analysis in leaves and fruit showed *C5-SD* expression was significantly reduced in VIGS-silenced plants (Fig. 9). Analysis of the *C5-SD*-silenced leaf and green fruit tissues of tomato showed a significant decrease in levels of *α*-tomatine. We anticipated that C5-SD could be catalyzing the conversion of cholesta-7-enol to 7-dehydrocholesterol in the cholesterol pathway^20^. Indeed, *C5-SD* silenced leaves showed accumulation of the predicted C5-SD cholesta-7-enol substrate while cholesterol and *α*-tomatine content was significantly reduced (Fig. 9). Levels of cycloartenol and the C-24 alkylated phytosterols intermediates, 24-methylenecycloartanol and isofucosterol were increased, whereas *β*-amyrin content was decreased in leaves. The decrease in *β*-amyrin levels in *C5-SD* silenced leaves is difficult to explain and might be through yet undescribed post-transcriptional control mechanism in triterpenoid biosynthesis.

In order to provide further evidence for C5-SD enzymatic activity, we performed yeast complementation assays. In *Saccharomyces cerevisiae*, the desaturase enzymatic activity is carried out by ERG3, which catalyzes the C5(6) desaturation of episterol to ergosta-5,7,24(28)-trienol in the synthesis of ergosterol, the main yeast sterol^43^. As cholesterol contains a C5-C6 double bond, a C5(6) desaturase would be required for its biosynthesis. To assess whether the tomato C5-SD was able to carry out this enzymatic reaction, we introduced the gene in a yeast *erg3* null strain, in which a kanamycin cassette replaced the native *ERG3* gene. *S. cerevisiae erg3* null mutants are viable, but are unable to synthesize ergosterol^43^. GC–MS analysis of organic extracts of the yeast *erg3* null strain confirmed its inability to accumulate ergosterol (Supplementary Fig. 5a). However, when expressing the tomato *C5-SD*, the ergosterol synthesis capacity of the *erg3* null strain was repaired (Supplementary Fig. 5a and b); indicating that C5-SD like ERG3, has the capacity to introduce a C5-C6 double bond into episterol (Supplementary Fig. 5c).

## Discussion

Alkaloids represent one of the three major classes of plants specialized (or secondary) metabolites with more than 20,000 reported in thousands of species to date^44^. The steroidal alkaloids produced by most members of the *Solanum* genus in the Solanaceae family are known primarily due to the toxicity of the major potato metabolites *α*-chaconine and *α*-solanine to mammals. To date, the research of SGAs was focused on structure elucidation, composition in different species and unraveling their biosynthetic pathway^5^,^6^,^8^,^10^,^16^,^17^,^18^,^45^. In this study, we identified an AP2/ERF-type transcription factor, which regulates the biosynthesis of steroidal alkaloids in tomato and potato, and likely in other Solanaceae plants producing SGAs (e.g., eggplant). It appears that GAME9 belongs to a separate clade of AP2/ERF transcription factors together with proteins regulating the biosynthesis of distinct alkaloid classes in other species namely, the pyridine alkaloid nicotine in tobacco and TIAs in *C. roseus*. This raises thought-provoking questions regarding the specificity of transcriptional regulation of alkaloids in plants and its molecular evolution (discussed below).

As in the case of its homolog *ERF189* located in the *NIC2*-locus in *Nicotiana tabacum*, the potato and tomato *GAME9* genes are positioned inside a cluster of similar, *GAME9-*like genes. In the tobacco *NIC2* locus, seven highly similar *ERF* genes were shown to regulate the expression of structural genes involved in nicotine biosynthesis^24^. When these *ERFs* genes were used to rescue nicotine content in a *nic2* background, they showed some functional redundancy. However, ERF189 was able to recover nicotine content to the wild-type levels^24^. Similarly, it appears that ORCA3, involved in regulation of the TIA biosynthesis, is also positioned inside a cluster of similar genes^46^. As we did not investigate the GAME9-like proteins, we cannot exclude functional redundancy between cluster members. Yet, *GAME9* was the only gene in this cluster that was co-expressed with other SGA genes and is thus likely to play a key role in the regulation of SGAs in both tomato and potato. Noticeably, in both species, only GAME9 and not the GAME9-like proteins have a serine-rich C-terminal domain (Supplementary Fig. 6). This domain was found to have a regulatory function in the *Catharanthus roseus* ORCA3 protein^47^ and could therefore serve to locate GAME9 primary homologs in different species. Nevertheless, while the *Catharanthus roseus*^47^ ORCA2 does not possess the serine-rich domain it was demonstrated to have an overlapping role with ORCA3. This suggests that the lack of the serine-rich domain in the potato and tomato GAME9-like genes does not exclude their possible function in the control of SGA biosynthesis.

The precursor for SGA biosynthesis is cholesterol, which undergoes several hydroxylation, oxidation, transamination and glycosylation steps to generate the SGA chemical diversity^3^,^5^,^48^. While still far from being resolved, cholesterol biosynthesis in plants is predicted to be a multi-step branch from cycloartenol. Recently, the first committed enzyme in the cholesterol pathway, SSR2, was described in potato and tomato^19^. Several studies demonstrated the tight crosstalk between the cholesterol and C-24 alkylsterol pathways in SGA-producing plants^19^,^21^,^35^. The SSR2 reaction is therefore a junction for controlling fluxes towards cholesterol and downstream to SGA biosynthesis. The enzyme SMT1, catalyzing the alternate branching reaction in which cycloartenol is trans-methylated to 24-methylenecycloartanol, is not less important in maintaining the balance between the two pathways^21^. Apart from *SSR2*, three additional genes including homologs of those encoding a *C5-SD*, *SMO1* and an *OXR* could be associated with cholesterol biosynthesis as their expression was affected very significantly in the tomato *GAME9*-altered transgenic lines. Our results showed that GAME9 is most likely involved in regulating *C5-SD* but is not associated with *SMT1* expression. Although we detected a significant increase in levels of the triterpenoid *β*-amyrin in potato leaves overexpressing *GAME9*, the transcript level of *TTS1* was not altered. This suggests either a different gene associated with *β*-amyrin biosynthesis in potato or a post-transcriptional mechanism for TTS1 activation.

Functional characterization by VIGS and yeast complementation assays, showed that C5-SD is an additional, currently the second enzyme reported to be involved in the cholesterol biosynthetic pathway. The desaturation reaction catalyzed by C5-SD is specific for the non-alkylated sterols branch, as shown by the accumulation of the cholesta-7-enol intermediate and decrease of cholesterol and *α*-tomatine. We speculate that there might be a different C5-SD paralog catalyzing the desaturation of C-24 alkylsterol intermediates leading to the biosynthesis of campesterol and *β*-sitosterol. Similarly Sawai *et al.*^19^ reported two paralogs, *SSR2* and *SSR1* involved in the biosynthesis of cholesterol and C-24 alkylsterols, respectively.

It appears that genes encoding enzymes in the mevalonate pathway, upstream of the SSR2-SMT1 branch point, are also under some level of control by the GAME9 transcription factor. This was evidenced in the tomato *GAME9*-Ox that showed a significant change in *HMGR*^35^ expression and significant decrease in *CAS* expression levels in the *GAME9*-RNAi lines. In potato *GAME9*-Ox lines, a significant increase in *CAS* but not in *HMGR* expression was observed. Additionally, when combined with SlMYC2, GAME9 could significantly transactivate the HMGR1 promoter. Expression of the gene encoding SQS, an enzyme downstream HMGR in the mevalonate pathway, was not altered in either the tomato or potato transgenic plants. Yet, it cannot be ruled out that altered expression of the mevalonate pathway genes (e.g., *CAS* and *HMGR*) may have been a result of a feedback mechanism (e.g., by SGA or cholesterol pathway metabolite intermediates) and not a direct regulatory effect of the GAME9 transcription factor.

It is hence apparent that GAME9 control of SGA biosynthesis is not restricted to the *GAME* genes of the core pathway between cholesterol and *α*-tomatine, but it includes the upstream biosynthetic genes of the cholesterol pathway and possibly upper in the pathway. This is likely crucial for ensuring the flux of precursors in times of SGA production and to maintain the homeostasis in the interface between the cholesterol pathway and essential phytosterol biosynthesis. Likewise, the *Catharanthus* ORCA3 was shown to activate several TIA biosynthetic genes as well as some primary metabolism genes involved in the synthesis of TIA precursors^22^.

It was previously reported that group IXa ERFs proteins from several plant species possess similar but diverse DNA-binding specificities and that each can differentially bind to multiple GC-rich sequences^32^. At least three different GC-rich boxes can be recognized in promoters of these transcription factors target genes: a P-box, a CS1 box and a GCC box. We performed transactivation assays by testing combinations of GAME9 and upstream regions of core SGA biosynthetic genes, mevalonate and cholesterol pathway genes as well as of some other genes altered in both *GAME9*-Ox and *GAME9*-RNAi tomato plants. The results suggested that GAME9 slightly activated the *C5-SD* gene upstream region containing a GCC-box.

**Figure 10:**
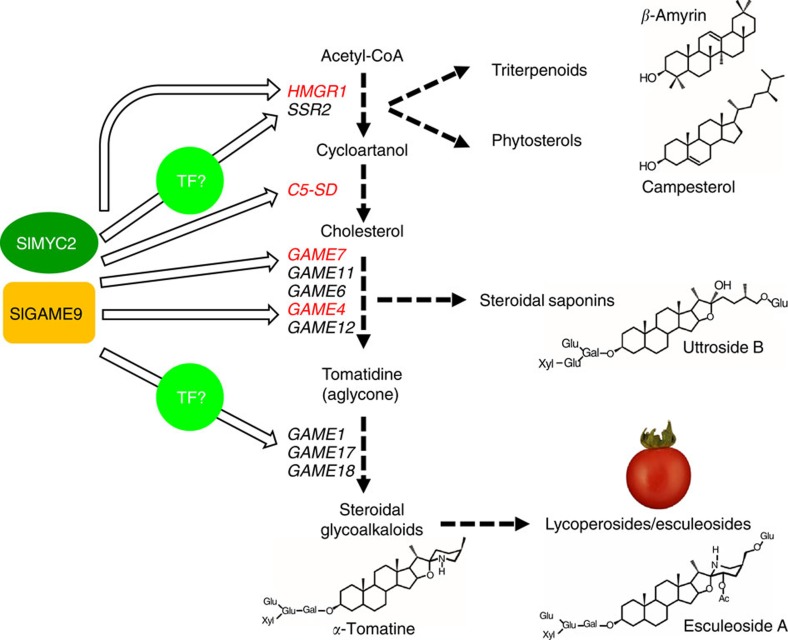
A model for GAME9 control of the steroidal alkaloid pathway and its precursors. GAME9 activates the synthesis of sterols precursors and SGAs in potato and tomato. GAME9 might activate genes across the pathway indirectly through an intermediate transcription factor (marked ‘TF?') or through co-binding with the SlMYC2 transcription factor. Promoter transactivation assays showed that GAME9/SlMYC2 might be partly regulating SGA biosynthesis by directly activating *HMGR1*, *C5-SD*, *GAME7* and *GAME4* marked in red.

Apart from acting directly on the *C5-SD* promoter, GAME9 might be acting indirectly through an intermediate transcription factor that by itself directly activates the promoters of core SGA genes (Fig. 10). In a different scenario, GAME9 requires an interacting factor and co-binding of both regulators to the promoter region of target genes in order to permit target gene activation. Such an interacting factor might be the SlMYC2 protein, a jasmonate signaling component shown to take part in activating the tobacco nicotine and *Catharanthus* TIA biosynthetic pathways together with the *NIC2* locus protein ERF189 and the ORCA3 protein, respectively. Our results support the control of tomato cholesterol (i.e., *C5-SD*), mevalonate (i.e., *HMGR1*) and SGA- related (i.e., *GAME7* and *GAME4*) genes promoters through co-transactivation by the GAME9 and SlMYC2 proteins. In the current model of transcriptional regulation of tobacco nicotine biosynthesis, when the bioactive jasmonate is perceived (i.e., JA-Ile) an active MYC2 is liberated^41^. The *NIC2* locus ERF proteins recognize the GCC-box and activate structural genes in cooperation with NtMYC2 that recognizes the G-box element in the same promoter. NtMYC2 also induces, directly or indirectly, the *NIC2* locus *ERF* genes (e.g., ERF189). In *Catharanthus*, MYC2 and ORCA3 factors likely act in a transcriptional cascade to regulate TIA biosynthetic genes and no evidence is available suggesting direct interaction of both proteins on the promoters of biosynthesis genes in a cooperative manner^30^. Similarly to the *NIC2* locus ERF proteins, the EMSA assays performed in our study showed that both GAME9 and SlMYC2 can recognize and bind specifically to GCC- and G-boxes present in their target genes.

The major potato SGAs are considered anti-nutritional factors for humans and their levels in tubers of commercial potato varieties are limited by law^3^,^4^. One approach to select low alkaloid potato lines is the identification of associated QTLs and carrying out marker-assisted selection. GAME9 is likely the gene underlying the major QTL on chromosome 1, reported by Sørensen *et al.*^36^ to explain 75% of the variance in SGA content among tubers in the population examined in the study. Hence, the identification of GAME9 provides a platform for the generation of Solanaceae crops with modified levels of SGAs. Furthermore, GAME9 provides a starting point for the elucidation of signaling and transcriptional regulatory networks that mediate constitutive and pathogen induced SGA biosynthesis in the Solanaceae.

## Methods

### Plant material and generation of transgenic plants

Tomato plants (*Solanum lycopersicum*) *cv*. MicroTom and potato (*Solanum tuberosum*) *cv.* Desiree were grown in a climate-controlled greenhouse at 24 °C during the day and 18 °C during night, with natural light. The *GAME9*-RNAi construct was created by introducing a *GAME9* fragment to pENTR/D-TOPO (Invitrogen) (by *Not*I and *Asc*I) and further transfer of the resulting plasmid to the pK7GWIWG2 (II) binary vector^49^ using Gateway LR Clonase II enzyme mix (Invitrogen). The *GAME9*-Ox constructs were generated by introducing the corresponding tomato and potato *GAME9* coding sequences into pDONR221 using the Gateway BP Clonase II enzyme mix (Invitrogen) and then transferred to the pJCV52 binary vector using Gateway LR Clonase II enzyme mix. Constructs were transformed into tomato and potato as described previously^5^,^8^. Primers used in this work are listed in Supplementary Table 6.

### Co-expression analyses

Co-expression analyses were done as described by Itkin *et al.*^5^ Briefly, the tomato *GAME9* (Solyc01g090340) and its potato ortholog (Sotub01g029510) were used as 'baits' in co-expression analyses, resulting in lists of co-expressed genes (*r*-value ≥0.8) for each bait, separately and shared homologs between the two species. The analyses were performed using tomato RNA-Seq transcriptome data from different tissues and organs (flesh, peel, seeds, roots, leaves, buds and flowers) and developmental stages (19 experiments in total)^8^ and potato RNA-Seq transcriptome data from different tissues and organs (40 experiments in total)^50^. The co-expression network was visualized with the Cytoscape program^51^.

### *In situ* RNA hybridization

*In situ* hybridization was performed as described by Hendelman *et al.*^52^ with minor modifications. The sense and anti-sense cRNA probes were produced by *in vitro* transcription with digoxigenin-11-UTP (Roche) using AmpliScribe T7 High Yield Transcription Kit (Epicentre Biotechnologies) from PCR fragments templates containing a T7 promoter sequence (tttgcggtaatacgactcactatagggcgaattgggtacc) flanking the sense/anti-sense *GAME9* full-length cDNA. Shoot apices from 13 days old tomato plants were fixed in PFA (3.8% PFA in 1xPBS, pH 7.0 by H_2_SO_4_), gradually transferred to ethanol and then to K-clear plus (Kaltek), and embedded in Paraplast Plus (Laica). Eight-micrometer-thick tissue sections were produced and mounted on Superfroset Plus slides (Thermo Scientific). Slides were treated successively with K-clear plus, an ethanol series, Diethylpyrocarbonate treated double distilled water, 2 × SSC, Proteinase K (1 μg/ml) in 100 mM Tris-HCl, pH 8.0, and 50 mM EDTA at 37 °C, Glycine (2 mg/ml) in PBS, two times with PBS, 4% paraformaldehyde in PBS, two times with PBS, triethanolamine (0.1 M, with stirring), two times with PBS, and increasing ethanol series up to 100% ethanol. For hybridization, slides were incubated with sense or antisense cRNA probes in hybridization buffer (0.3 M NaCl, 10 mM Tris-HCl, pH 8.0, 10 mM sodium phosphate buffer pH 6.8, 5 mM EDTA, 50% v/v deionized formamide, 10% w/v dextran sulfate, 1 × Denhardt's solution, 200 μg tRNA) overnight at 55 °C. Following hybridization, slides were washed successively twice with 0.2 × SSC at 55 °C. Then, slides were blocked with 1% fresh Boehringer block (Roche) in 100 mM Tris-HCl, pH 7.5, and 150 mM NaCl, and then with 1% BSA solution (1% BSA, 100 mM Tris-HCl, pH 7.5, 150 mM NaCl, and 0.3% Triton X-100). Blocked slides were incubated with antidigoxigenin antibodies (Roche) for 2 h at room temperature and then washed three times with 1% BSA solution and three times with detection buffer (100 mM Tris-HCl, pH 9.5, and 100 mM NaCl). Then the slides were incubated with NBT/BCIP color development substrate (Promega) for 24 h and then washed with double distilled water followed by increasing ethanol series and then mounted and analyzed. The expression pattern detected by the *GAME9* antisense probe was compared with a control *GAME9* sense probe, which showed only background signal.

### Phylogenetic analysis

A literature search was performed to identify functionally characterized proteins belonging to the ERF family of transcription factors. Amino acid sequences were aligned using ClustalW2 (ref. 53). A phylogenetic tree was built using the neighbor-joining method^54^ implemented in MEGA6 (ref. 55). The analysis involved 50 amino acid sequences and evolutionary distances are in units of number of amino acid substitutions per site. All positions containing gaps and missing data were eliminated. Accession numbers for sequence data used in this tree can be found in Supplementary Table 8.

### Preparation of plant extracts and metabolite analysis

Profiling of phytosterols was performed with three biological replicates (i.e., three plants for each genotype, *n*=3) with each plant being one independent extraction and was carried out as described previously^8^. Briefly, 100 mg frozen leaf powder was extracted at 75 °C for 60 min with 4 ml chloroform/methanol (2:1 v/v) containing epicholesterol as an internal standard. Extracts were kept at room temperature for 1 h, solvents were evaporated to dryness, and the residues were saponified at 90 °C for 60 min in 2 ml 6% (w/v) KOH in methanol. Upon cooling to room temperature, 1 ml n-hexane and 1 ml water were added, and the mixture was shaken vigorously. Following centrifugation to separate the phases, the hexane phase was transferred and evaporated to dryness. Subsequently, 50 μl of N-methyl-N-(trimethylsilyl) trifluoroacetamide (MSTFA) was added, the sample was shaken vigorously, and the mixture was transferred to an autosampler glass vial with a 100 μl conical glass insert and analyzed by GC–MS according to Itkin *et al.*^8^ Compounds were identified by comparison of their retention time and mass spectrum to those generated for authentic standards analyzed on the same instrument. Preparation of extracts for SGAs analysis was performed as in Itkin *et al.*^8^ with three biological replicates (*n*=3) and the following modifications: potato and tomato extracts were diluted 80 and 50-fold, respectively, before injection. Compounds were analyzed in MRM positive mode using a UPLC-TQ-MS (Waters), equipped with Acquity BEH C18 column and Triple Quadrupole MS detector. Mobile phases A and B, column temperature and flow rate were set as described previously^9^. For potato samples, *α*-solanine and *α*-chaconine were isocratically eluted at 20% B for 10.5 min, the column washed with 100%B for 3.5 min and re-equilibrated at 20% B for 1 min. The following MS parameters were applied: capillary voltage 2.7 kV, cone—61 V, collision—65 eV. Relative quantification was done using the TargetLynx program (Waters), using the sum of two MRM transitions for *α*-solanine (868.5>398.4, 868.5>706.5) and *α*-chaconine (852.5>398.4, 852.5>706.5). For tomato samples, the following linear gradient was applied for *α*-tomatine analysis: 15 to 30% B over 5 min, 30 to 50% B over 10.5 min, 50 to 100% B over 0.5 min, held at 100% B for a further 1.5 min, then returned to the initial conditions (15% B) in 0.2 min and conditioning at 15% B for 1.3 min. MS parameters: capillary—2.72 kV, cone—60 V, collision energy—40 eV. MRM transitions were set as 1034.5>416.3 and 1034.5>578.3. The first transition trace was used for *α*-tomatine quantification.

### Quantitative real-time PCR

Gene expression analysis was performed with three biological replicates (*n*=3) for each genotype. RNA isolation was performed by the Trizol method (Sigma-Aldrich). DNase I (Sigma-Aldrich)-treated RNA was reverse transcribed using a high-capacity cDNA reverse transcription kit (Applied Biosystems). Gene-specific oligonucleotides were designed with Primer Express 2 software (Applied Biosystems). The *TIP41* gene^56^ was used as an endogenous control for tomato samples and the *NAC* gene^21^ was used for potato. Oligonucleotides used are listed in Supplementary Table 6.

### RNA-Seq library preparation and sequencing

RNA-Seq libraries were prepared as described by Zhong *et al.*^57^ with minor modifications. Briefly, 5 μg of total RNA was used for poly(A) RNA capture using Dynabeads Oligo (dT)_25_ (Invitrogen), fragmented at 94 °C for 5 minutes and eluted. The first-strand cDNA was synthesized using reverse transcriptase SuperScript III (Invitrogen) with random primers and dNTP, whereas the second-strand cDNA was generated using DNA polymerase I (Enzymatics) using dUTP. After end-repair (Enzymatics), dA-tailing with Klenow 3′-5′ (Enzymatics) and adapter ligation (Quick T4 DNA Ligase, NEB), the dUTP-containing second-strand was digested by uracil DNA glycosylase (Enzymatics). The resulting first-strand adaptor-ligated cDNA was used for PCR enrichment (NEBNext High-Fidelity PCR Master Mix, NEB) for 14 cycles. Indexed libraries were pooled and sequenced.

### Transient expression assays

Transient expression assays in *Nicotiana tabacum* protoplasts were performed as described previously^23^,^58^. Briefly, protoplasts prepared from tobacco Bright Yellow-2 (BY-2) cells were transfected with three different plasmids. The first plasmid (reporter plasmid) contained the firefly luciferase (fLUC) gene under control of the investigated promoter; the second plasmid (effector plasmid) contained the ERF transcription factor GAME9 or SlMYC2 driven by the cauliflower mosaic virus 35S promoter (pCaMV35S) and the third plasmid (normalizer plasmid) contained the renilla luciferase (rLUC) under pCaMV35S control. After transfection and overnight incubation, the protoplasts were lyzed and both fLUC and rLUC activities were measured with the Dual-Luciferase Reporter Assay System (Promega). The fLUC activity is a measure of the activity of the investigated promoter, whereas the rLUC activity reflects the transfection efficiency. For normalization, the fLUC value of each independent transfection was divided by the corresponding rLUC value. For screening and confirmation experiments, 4 and 8 transfections were performed for each promoter-GAME9/SlMYC2 combination, respectively, and the obtained normalized fLUC values were averaged and compared relative to the values obtained from transfections with an effector plasmid containing the GUS gene.

### C5-SD promoter cloning

C5-SD promoter deletion constructs were PCR-amplified from the original full-length C5-SD promoter construct using the primers listed in Supplementary Table 6. Promoter fragments in which the CACGTG motif of the G-box and the AGCCTGCCAC motif of the putative GCC-box were mutated into ATGTGA and GATTACAGTC, respectively, were generated by overlap extension PCR using the primers listed in Supplementary Table 6. The obtained PCR products were directly recombined into pGWL7 using single step BP/LR combined Gateway reactions and the resulting reporter constructs were sequence verified before being used for transient expression assays.

### Electrophoretic mobility shift assays

The coding sequence of GAME9 (amino acid 40–219) and SlMYC2 were cloned in pET28 vectors. The recombinant proteins with a His-tag were expressed in *E. coli* BL21 Star (DE3) (Invitrogen) and affinity purified. Unlabeled oligonucleotides containing GCC-, G-boxes and their mutated versions (shown in Supplementary Table 7) were used to determine *in vitro* binding. Annealing of the probes was performed by boiling equimolar concentration of sense and antisense oligonucleotides to 95 °C for 5 min and cooling to room temperature. Electrophoretic mobility shift assay was performed as described previously^59^ with 200 ng of protein and equimolar concentration of probes. The probes were separated in 1% agarose gel. The uncropped gel image is shown in Supplementary Fig. 7.

### Virus induced gene silencing

Vector containing a fragment of *C5-SD* gene was generated and VIGS experiments were conducted as described previously^5^. The infection was performed in the background of a transgenic tomato line expressing the *Antirrhinum majus* DELILA and ROSEA1 (DEL/ROS) transcription factors, that convey a purple anthocyanin-rich phenotype to the fruit^60^. The VIGS vector includes the candidate gene as well as the DEL/ROS sequences in a way that allows locating leaf or fruit green patches in which the candidate gene was likely silenced. Plants infected with Agrobacterium, containing empty vector and helper vector pTRV1, were used as control. Leaves and green fruits were collected after 4 and 6 weeks post-infection respectively, and analyzed by LC-MS and GC–MS as described before. Oligonucleotides used to prepare the pTRV2 vector are listed in Supplementary Table 6.

### Yeast complementation assay

*C5-SD* was amplified with primers listed in Supplementary Table 6 and cloned into pDONR221 by Gateway recombination. For expression in yeast, the entry clone was recombined with the destination vector pAG423GPD-ccdB (Addgene plasmid 14150)^61^ yielding the pAG423GPD-(C5-SD) expression clone. *S. cerevisiae erg3* null strain was obtained from EUROSCARF (accession number Y12667; genotype: BY4742; MATα; his3Δ1; leu2Δ0; ura3Δ0; YMR056w::kanMX4)^62^ and cultivated on yeast extract peptone dextrose medium (Clontech) supplemented with 200 μg/ml of G-418 disulfate (Duchefa). This yeast strain was transformed with the pAG423GPD-(C5-SD) expression clone for complementation or with the unrecombined destination vector pAG423GPD-ccdB as a control. Transformants were selected on plates containing synthetic-defined (SD) medium with the -His dropout supplement (Clontech). For each strain, 5 individual colonies were used to inoculate 5 ml of liquid SD-His medium. The cultures were grown for 2 days at 30 °C with shaking at 250 rpm after which the yeast cells were collected by centrifugation. The yeast cells were lyzed by adding equal amounts of 40% (w/v) KOH and 50% (v/v) ethanol to a final volume of 1 ml, followed by boiling for 2 h. Sterols were extracted from the lyzed cells by liquid-liquid extraction using three times 500 μl of hexane. The organic phases were pooled, vaporized to dryness and trimethylsilylated with 10 μl of pyridine and 50 μl of N-Methyl-N-(trimethylsilyl) trifluoroacetamide (Sigma-Aldrich) for GC–MS analysis. GC–MS analysis was carried out as previously described^63^.

## Additional information

**Accession codes:** Tomato RNA-seq data associated with this manuscript have been deposited into the NCBI Sequence Read Archive with BioProject ID PRJNA307656.

**How to cite this article:** Cárdenas, P. D. *et al.* GAME9 regulates the biosynthesis of steroidal alkaloids and upstream isoprenoids in the plant mevalonate pathway. *Nat. Commun.* 7:10654 doi: 10.1038/ncomms10654 (2016).

## Supplementary Material

Supplementary Information Supplementary Figures 1-7 and Supplementary Tables 1-8

Supplementary Data 1 Detailed list of genes co-expressed with GAME9 in tomato, potato and r-values for specific genes involved in the synthesis of SGAs and sterol precursors.

Supplementary Data 2 Detailed list of genes showing altered expression in GAME9-RNAi (tomato) and GAME9-Ox (tomato and potato) lines. Common set of genes found in GAME9-Ox
