# Supplementary material for: GAME9 regulates the biosynthesis of steroidal alkaloids and upstream isoprenoids in the plant mevalonate pathway
Source: Nat Commun. 2016 Feb 15;7:10654. doi: 10.1038/ncomms10654 (PMC4756317; doi:10.1038/ncomms10654)
Supplement: Supplementary Information — Supplementary Figures 1-7 and Supplementary Tables 1-8 [file ncomms10654-s1.pdf]

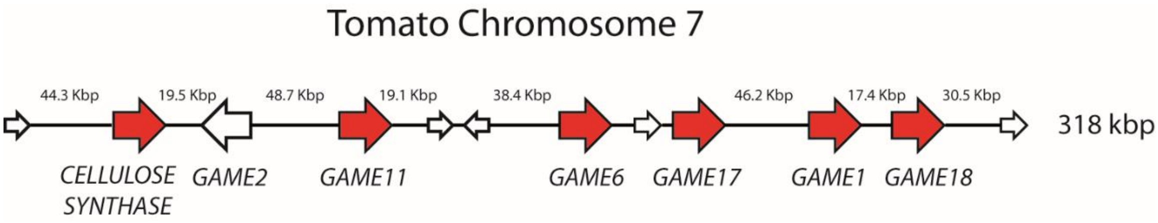

Supplementary Figure 1. Schematic map of altered SGA-related genes in tomato *GAME9*-RNAi and *GAME9*-Ox transcriptome.

Among a set of 27 common genes in the *GAME9*-RNAi and *GAME9*-Ox tomato lines, we found a significant representation of SGA biosynthetic genes (*GAME'*s) located in chromosome 7 (*GAME*'s- *1*, *6*, *11*, *17* and *18*) and a *CELLULOSE SYNTHASE*. Altered genes in the region (spanning ~318 kbp) are highlighted in red.

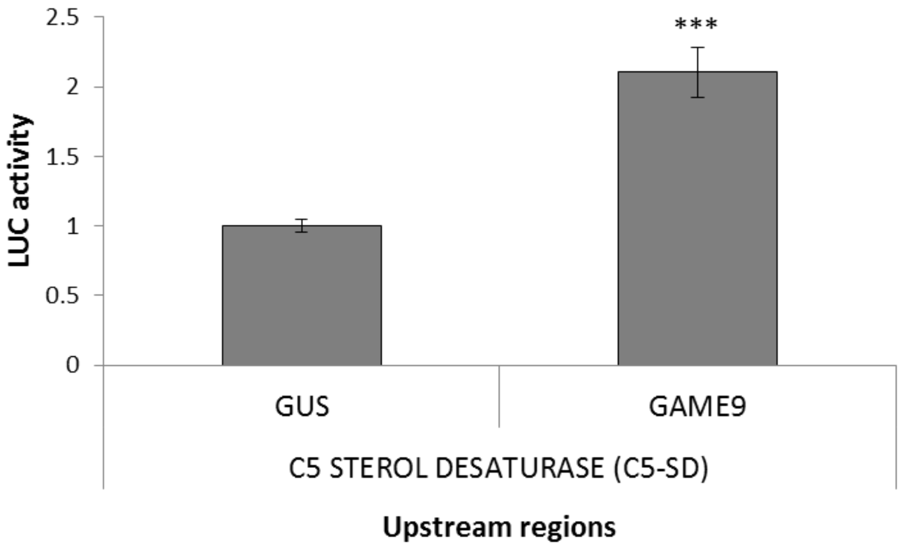

Supplementary Figure 2. Validation of transactivation assays of putative downstream gene promoters by the GAME9 transcription factor.

Validation experiment (n=8) was performed, confirming the transactivation of the *C5-SD* promoter. Values in the y-axis are normalized fold-changes relative to protoplasts cotransfected with the reporter constructs and a pCaMV35S:GUS (GUS) control plasmid. For the normalization procedure, see Materials and Methods. Details for each promoter are provided in Supplementary Table 7. Student's *t*-test was used to assess whether the transgenic lines significantly differ from wild-type plants: ***P value < 0.001.

Δ(7)-STEROL-C5(6)-DESATURASE (Solyc02g086180)

>SL2.50ch02:48924609..48928542
CACCGCCGTGGAAGTTTATTCACGGATATTACCACGAACTATTAATTTCTCTCTCTAGATCTCTCATCTCTCTCTCAAAAATTCTTATATTTTTCATTTA
TCAAGTGTGTGTGAATTCGATCCTAACAACTATACGAGTAGAACTACCTAACATTTCAAGTAGCGGCATGAAATGTCTATTTAGGCTAAAAAACAAA
GGACCACGAGGAAAATAACAACGGAGAGAGTGATTTTATAGCCCCTCAGCCAAATAAATAAATAAATAAATAAATTCTCTTTGGCCGGCCAGCTAG
TTTCAATCTTCGCAAAATAATAGATATTTCTTGATAAGAAGGTTTAGATCCATAGCATAGGTAACATGAGAAATATGAAAATTAATATCATATAATAT
ATTTAATTTAATTTTGTAAATTGAATCTACAAAATAATGATGTATGCACTATTTAATATTTTGTGAATGTTGAAATATTGTTTCATTAGACTGATATGA
TAGATTGCTAAACTAAATCTCATAATATATATTACATTAAAGCTCAAACTATAATCTCTTTGATACTATTTCCATAGAACGAATGTGCATTAACAGAG
GGGAAAGGAGAAAGACCACATAATGATTAATCATAGAGATGATAATAAAACCAACAAAAAAAAAAAAAAAAAGACAAAGTAGAACAAGCCAAACT
TATGAAGGCCATAATAACCATATAATAAAACAGACAAAATTTTAATTTTTGAATTTTTTTAATGTAGTCGAGTTTCCCCCCTTCAAAATTAATTATTGT
GACATCAATATACTGCAACAAGTGGCTGGTTAGTTCAACACAAGTTTAAAGGAATAACACAAACAAACTTTCATCTAGTTATATCGTTTTAGTAAAA
TGGACATAAGTTATTGCTTAAATTCCATAACTGTTTATATTTTCTTAAAAGTTTGATACATTTGTTTATGAAAATCACTTAATAGCTTAATGTTAGATG
TATACATATCACAATTTTAGCTAATATCAAAGTTAATTTTGTGTCTAACTTTTTTATATATATCTACTCGATTTTCAATAGTATCTAGATTAATGAAACA
TGACAAGGTGAATTCAAACTAAGGATTAAAATTAATAAAGCATGATAAGATTATATTAAATATATTATTGTTATCAGTAATATTTTCTCGATTTATTA
TTACATAATTATTAATAAATGTATGAAATATTACTCTAAAAAAAAAAGTGATTGAATGATAAAAATAAAATGTGAAATCTATCATATTTTTAAAAATAT
TAAACAAATAAAATCAAACGAACGATCTTATTAGAATCTTTTGCCAGTTTAGCTCACCACACAACACGTGGGAGTAGTAGTCCAAAATGGAATCAAT
TTTCATTAAATACAGCCTGCCACACATGCATATATTGTGTTACCGCACTAGCCCCTCTTTGTTTTCCTCTCTATCTTCACTCCATAGTTCCTCCTTTTCCG
GCGGACCATCGCCGCCGGAGTCTGGTAAAACCCGACCCGAGACGGACATATTTCTCTTTTACCTAGTATACCGTCGGAGATGGAGGATTACTTGAA
GCTATTCGTGGAGGAGACATCGTTTTACAACCGTCTGGTTTTGGGTACATTCTTGCCGGAATCATGGTGGGGACCACTTCCTCATATGCTTCAAGGA
TGGCTCCGTAACTACATTGGCGGTGTTTTACTTTACTTCATCTCCGGTTTCCTCTGGTGCTTCTATATTTATCACTTGAAACGCAATGTCTATATTCCCA
AAGATGCCATACCATCAAAGCAAGCAATGCTGTTGCAAATATCAGTTGCTATGAAAGCTATGCCGTGGTACTGTGCCCTTCCATCACTTTCTGAGTA
CATGATTGAAAACGGATGGACCAAATGTTTTGCGAGAATAAGTGATGTTGGATGGCCTACCTACATCATCAATGCGGCTATTTATCTTGTAATAGTG
GAGTTTGGAATCTACTGGATGCACAAGTTATTGCATGACATAAAACCTCTGTATAAATATCTGCATGCTACACATCATATTTACAACAAGCAAAACA
CACTTTCCCCATTTGCTGGATTGGCATTCCACCCATTGGATGGAATACTGCAGGCAGTGCCACACGTTGTAGCTCTTTTCTTGGTGCCAATGCATTTC
ACTACACACATAGTGCTCATATTCGTGGAAGCCTTATGGACGGCTAATATTCATGACTGCATACATGGTAAGGTGTGGCCTGTAATGGGTGCCGGT
TATCATACCATTCACCATACAACATACCGCCATAATTATGGTCATTACACAATATGGATGGACTGGATGTTTGGAACTCTTCGTGATCCCGTTGAAGA
GGATGCCAAGAAAATGTAATGTGCATGATTTGCACTCAAGGATGTTCCTTATTGTTCATTGTCCCAGCAATTTATCTCGTGTTTCCATCGTTGGTGGA
TGTAAATTTTGATGTAGTAGTTTATGTGAAAACAATTTGCTTTTATTGTCTTTTCTTCCCCTATATATTTAATAATAAGCAATAAAATCTGTGGGGAAG
AAAATTTCAGTTTCTAACTTTATGCCGGAGTCCTTTGAGGTAATCATATAACGAAAGCTAAATGTGAATCACATTGTCACCATGTTATGGTGGATA

Supplementary Figure 3. G-boxes and GC-rich motifs found in the promoter of the *C-5 SD* gene.

Three GC-rich boxes in the promoters of target genes are known to be bound by group IXa ERFs (Shoji et al., 2013). A P-box recognized by all ERFs, except clade 1 AtERF1; a CS1-box bound only by clade 2-3 ERFs (e.g. ERF163, ORCA3, AtERF13) and a GCC-box bound by all, except clade 2-1 ERFs (e.g. ERF189, ERF115). The presence of G- (blue), P- (green), GC-rich boxes (yellow) and the 5'UTR (pink) present in the promoter construct used in the transactivation assays (~1,550 bp), and the coding sequence (red) are highlighted.

GAME4: CYP88 (Solyc12g006460)

>SL2.50ch12:941286..934011
GAGACTTGAACCTTCCTCTTTCGTTGGGCTTCCAAATTAATGCAATCTATTCGCATAAATAATCACAATGAGATTTCTAAACTAACAACATCCATAAT
ACTACATGTATATCCTAGACCTAAAACCTCACCAAATTCTATAAAGTTTCTAATCTTGATGCCAATTAATACGTCAATTCGCATAAATTTTAAATTTTA
AGCTTAGAGTTGAGTCTCAACTTTCTCCAAATATAATATAATTAGTGATATTCATATAATATAATACATCTAATATTTAATTAGATATCTCAATATATT
AAACTTAATTAACATGTGATAACATATAAAAAAAAACATCAAAACCCCATGTGAAGTCTGGCGCTAATCTACCACCAAAACAAATATGCATAATTTT
CTTGCCCTAATTGAAATAATATGACATCATTCCTTAAAAAAATTGACAATCACCATGCTATGATTGCAAACCAATGTGATCTTGTGAACAATAAAATT
AATATATAATTTTTAGCGGTAATAAATAAATATATTAATAAAAAATGTTAAAATCTTTATCGGTATTAATTAAATGTCATTAGATCCAATATATTTAAA
GCCTTTAGGTCATATAAAATGAGTATTAAATGCCATTAAAAATGAAAATTTAACAATAATTTCTAATTAATTACCGCTAAAAATTCACACAAAAATAA
ACAATTGAATAGCCCATTGCTCGAATACGGTGCTTTACTCCAAGTCACACTTAACTCCATTTAATTTGATTCACTTATACCTATGGGTCAAAGTGATA
GAGGATATTAAAAACCAACTGAAATAATTATTAAAAAAATACTCATCAACTAAATATTTTTAGTCAAACTTGAATAATTTAAATTTTTAAATCAACTTT
ATTGACTAATCAAAATGACTCGGGACAACTATCGAAAGAAATAAAAATATTTATTTTTGAATTTTTTTGAAAATGGCCTTTCGAGTCGTTATATTTAG
ACCAATAACAAAAAAAAATCACAAAAAGCAATAAACATAACATTTGATTGTTTCTTTTATTTTTTTAATTAATAATATTTGCATAAATTATGTGCAAAA
TTTTATATTATTTTACAGAAGGATATAATATGGTATGAAAAATGGGTTTTATAAGTAAATATGCTTAAAAGCTAAAATAACAAAATTAATAGTGTGA
TTGACTAAATTTTTTTAGAAAAATATTATAGAAAATTACTTTTTATCAAAATATTTTTGTATATTAACAATTTTTGTTGACTAATTTTTTTTTTTTGATTAA
TATTAGAGTTCTATTAGTGCTTGATCAAGATTTCAAAATTAATTTTGTTGTTCCAATGATAAAATGTGATTGATATTTTAAACTATTTAAATAGTTTTTT
TTGAATCTGATACTACTAATATTAAATTTAAAGCCAACAACCCCGCAAAATAAATCTTCACGTGACCAATGCATCAAGTGGAATTTATTAAAATTAAA
TACGACAAGATATGTAGAAAATTATTGTTACAAAAATTAGAAAGAAACAAAGTCCATCTTTGAATTCTCTCACATAAAATATTGGGACCCAACGTTA
GAAACATTATTGTTATGGCAAAAGAAAAAATCGGTTATCATCGACGTATCTGATCCAGTAGATAAGATTATTTCTCTCGTTAATCGTAGGTTTTGAAT
TTGATTTTTTAGTATTAAAAAAAATTTTTGGTAAGAAATGTCTTTCCCTATCCTTCTAATGGAATCCTATGTAGCGCGAATTCGAAATAGTCTAATTTC
AATTTAATTATTAAACACAAAATGAAAATAAAAACAAAAATCCTCATTTTACTAATAATCATAATAATGATGTAGAAATTATTAGCTAATCCCAAAAT
ATTAATCATAAATAAAATTTAACTAATAATCAAAATAAATATTATATATAAACCAACAATCAAAACAAAAACTCATAGTATAAGACAGTACAACAAG
TGTAAAGGTTGTGTAAAAGCTTTTAGGTTTCTAGTCAAATAACTATGATTTTTCTTCAAAAATATCTTAAAGAATTATATATATAATCAATATATGATT
TTTCATTTTTTTTGGTTTATTATTATTTTAGTTTTTCCCTTTCTTTTAATTTTTTCCATACCTTTTGTCCGTAATCATGGACCATATCATTATTTGTTAACTG
AAATGACAAAAATAAACCAAAATTTAACAGCTAACATAAATAAGTTTTTCTCAAAATTCAACAACACATTTAAACCTTTTCATTTAAAAAAAAAACAAT
TTCTTTAATAATTTTACAAAATCGAAAATATATTTAGTTTATCAATAATGAAACCTTTTAACGAAAGTTGGGGGGCTAAATTATATTCACGTGATTGT
ATTTTTTTTTTTTAATTGCACTTCTATTAAGGAATAAAATTATAAAGATTTAACTATCTTTGAAAGTAAAAAGGTTAGTATTGTCTTTTCACATATTTGGT
CTCATCATTATTGTTGCCATATTTTTTTAGATAAATAAATGTAGCCATTTACTACCTCCAATCAAATTCACAAATCCATCCAAATTATTCAAAGAAAAA
AAAATAGAGAGAGAGAACAATGGATTTCTACAATTTAGCCTTGTTCTTCATAGCTTTAATACTTGGAATTTTCACATTTTATGCCATATTAATGAGAA
TAAATGGTTGGTATTATGCAATCAAATTTTGTTCAAACAAATATAACATCCCAAATGGTTATATGGGTTTGCCATATTTGGTAACACACTTTCTTACT
TCAAAGCTTCAATGTGTGGTGATCCAAAATCATTCATTGATTTCTTTGCTACTAGGTTTGGAGAAGGAGGAATGTATAGGGCATACATATTTGGGAA
GCCAACAATTATGGTGACAAAGCCAGAAATAATTAGAAAAGTTTTGATGGATGAAGAGTATCTTGAAAGAGGTTTGCCTAATTATATGAAAAAATT
AATTGGATTAACAACTTCGATAGAAGAAGACAAATATTTTCGTAGATTAACAGCACCAGTAAAAAGTCATGGATTATTATCTGATTATTTCGATTAT
ATCGATAAAACTGTGAGTTCTACATTAGAGAAATACGCTACTACGGAAGAACCTGTTGAGTTTCTTCATAAAATGCACAAGCTTACGTTTGAGGTGT
TTATGAGACTTTTAATTGGTGATGAAGTTAATCAAGAATTATTTGATGAAATGTTTGAGGAGATTACTGCTGTAATTAGTGGTGTTCACAATTTGCC
AATTAATCTCCCAGGATTTGCTTATCATAAGGGACTCAAGGCTCGAAAAGTACTAGGAGAGGTATTTAAAAAATTAATTGATGAAAGAAGAGAAGC
CATGAAGGATGGAAAATCAATGCCAAAGGCAAACATAATTGATATGTTGTTATCAAACAACAATCAAGATTATGAAGCAAACATGTTGAGTGACAA
GAAGATCATTGAAATCCTAGTTTTGTTTTCATTTGCTGGTTTTGAACCTGTTGCTCTTATGTCTGTCAAGGCAATTTTCCACTTACAAAAACATCCACA
TTTCTTGGAAAAAGCCAAAGAGGAACAAGAGGAAATAGTAAAGAGAAGAGCATCTTCAAATGCTGGACTTAGTTTTGATGAAATTAGACAAATGA
CATTTGTTAGTAAGATAATTAATGAAACGTTACGTATAGCTACTGATCAGTCGGTATTCCTTAGAGACACAAGTACTACTTTTAACATAAATGGGTAC
ACCATACCCAAAGGGTGGAAGTTTTTTGCAGTTGTATGGAATATTCATATGAATCCTGATGTTTATGTTCAACCTAAGGAATTTAATCCTTCGAGATG
GGATGATATTGAAACTAAGCCAGGCATTTTTCTACCTTTTTCAATGGGCCCCAAATCATGCCCAGGATCCAATTTGGCCAAGCTTCAAATTTCAGTAA
TTCTTCATTATTATCTTCTTCACTACAGGGTTGAGCAAATTAATCCAGAGGCTAGATGTTATCCTCCTGAAAATTGTCTTGTGAAATTCAAGAAGCTA
TCGATCTCTAGTAATGGTAATTAA

GAME7: CYP72 (Solyc07g062520)

>SL2.50ch07:65217385..65223561
GGTACAAGGACTAGCAGCCTATGAGGAAGACAAATGGGCCAAACATAGAAAAATTATCAATCCCGCTTTCCATCTAGAGAAGCTAAAGGTTTGTCA
GACGTTTCTTCGTTTTTCCTCCTTTAACGTATGACTATTTCTAGTAAATTCATCATTTGTCAAGACTCTTCTACAGTTCTATGTAAGTTCATAGGCTTGG
ACGGGGCATTTAGTCTATGTAAGTTGATAATGTCTTAGATATATTACCGTAAAATGAATCTTTTTTATATTAAAGAGATTTGTAATATTGCTGAACGT
GTTTCACCTTAATTGTTGTTTGAACGCTCTTTCTTTTTATTAAACTAGATGGTGATGGTATCACTATATCTGTATTCCTAATTCTGTTTCAAGTGTTGTG
TCATCGTTATATGTTCCCTTACATCATTTTCTCGAACAGCATATGCTTCCAGCTTTTCACTTGAGTTGTAGTGAGATGTTGAGTAAATGGGAAGACGC
TGTTGAAGGCTCTTGTGAGATAGATGTATGGCCTCACCTTCAGCAATTAACTTGTGATGTGATCTCTCGTACAGCTTTTGGTAGTAGTTACGAAGAA
GGTAGAAAGATTTTTGAACTTCAAAGGGAACAAGCTCAGCATTTTATCGACACTGTACGCTCAGTTAATATCCCAAGATGGAGGTAAATTAATTTGC
CTGAGAATCGAAAAATTGTAGCCAAAGCTATGTTGTTCGGACTCTTAAAAATGATGATGGTTGCATGTTGGATCTTCCAAATATTAGTGCATTTCTA
TAGAATCCGACATGGTTGCAGGAACTATTTTGGAGAGTCCGAACAACATAGAACCAAAGTTCATCAGGAGATGCACATAGCTTTTACTTCATCTAAT
GCATTTTGGTTTTCTGTCTTATAATATAATTTCAAAGATTTTTGCCAACAAAGAGGAACAGAAGAATGAAGGAAATAAAAAACGAGGTTCGGACCTC
AATGAAAGGTATTATCGATAAAAGAATGAAGGCAATGAAAGCAGGTCATGATGCCGATAATGAGGATCTGTTAGGCGTATTGCTTGAATCAAATTT
CAAAGAAATTGAACAGCACGGAAACAAGGATTTTGGAATGAGCATCGAAGAAGTCATCGAAGAATGCAAGTTATTCTATTTTGCTGGCCAAGAAA
CTATAGCAGTCTTACTCGTGTGGACTCTGGTGTTGTTAAGTAGGTATCAAGATTGGCAGACACAGGCCAGAGAAGAAGTCTTGCAAGTGTTTGGGA
GTCGGAAACCAGATTTTGATGGATTAAATCATCTAAAAGTTGTGAGTATTCTGTTTTTTTTAATTCTTGGTTTAGTTCATGTGCCATGAGTTCCTTCTC
TCATTGTTATTGCTATTGCTTGAGAGCTTTGTGATACTAATGTTTCATTGTCCACGCACCAGATTCTAAGCACTGGGCGTGAGGTGGGGTGTTAAAA
GATGGAGCAGATTCCTTATAAAGCTTGGTCAATCCTCCTCTCTTTGAGCTAGCTTTTGCGGTGTGAGTTAGACCTAAGACCTAATTTCACAATTACAA
CTTGTAACTTACAAGCTAACGATGATCTTGTACGAGTCATTAGATATAACTGAAGAAGAAGCCTTTTTTTTGCAGGTAACGATGATCTTGTACGAGT

CACTAAGGCTATATTCCCCATTATTTACACATAACAGACAGGCAAGTGAAGACACAGTATTAGTAGAATTGTGTCTACCAGCTGATGTGCTAGTTTC
ATTGCCCACGATCATATTGCATCATGACAAAGATATATGGGGTGAAGACGCAAACAAGTTCAATCCAGAGAGATTTAGAGAAGGAATATCAAGTG
CAACAATGGGTAAAGTTACATATTTTCCATTTTCATGGGGTCCAAGAATATGCATTGGACAAAATTTTGCAATGTTAGAGGCAAAGATGACTCTCTG
TATGATCCTACAAAGCTTCTCTTTTGAATTGTCTCCATCCTACACACATGCGCCTCAACCCCTACTTACCATTCATCCTCAGTATGGTGCTCCTCTTATT
TTCCACAAACTATAATTTCATATTACGTGAGGTATCTTTCACTTGTACGACTATACTGGATATGTTATTGTTCAGACAAACTCAATAACTCTTGTTTAA
ACCTTTTATCTGTACCAGAGAATAAATAACATGTTGCAAATTATATACAAATCATCTCAAGAACCAATGAGAAGCTTATTGTTATGACAAATTTAGAT
ATATTCTACAAACACATTAAGCTAAAGAATTTGTGGAAAAAATAACTGTTTTTTTTTTGGTCACAATAAAAACATGTGACATGCTAGTAAGCAGTAA
GCACAGCCGGCCTTTTTGGCAATGAAACCACTACATACACTTGTACACTAATTCTTTTTTCATTTTTTAAACTAAAATTATGAATGTCAGAATACGAAA
TAACAAGTAAAAACAAACGAAAATGATTAAAAATATAAGAGAAAATCAGAGCCACTCATTGGTGTCTTTGCTCATCATTGTTGACGACGTCGTTTGT
TAGTTCCCAGAGACATAGTATATATAAATTAATACTCCTCGTTTTTTACACACAAATTCAACTCAAACAAAATGGACGAAATTCAAATATTAGTAAGA
GTGTGTTGTTCTGCCATAGCAATTGCTCTGTTTGTGTGTCTGTGGAAAGTACTAAATTGGGTTTGGCTCAATCCGAAGAAGTTGGAGAATTTGTTGA
GGAAACAAGGGCTAAATGGGAACTCTTACAAAATATTGTATGGGGATTTGAATGATTTTATTGGAATGATTAGGGAAGCTAGTTCAAAACCTATGA
ATTTGTCTGATGATATAGCTCCAAGATTGGTGCCTTTCTTTCTTGAGACCACCAAGAAATATGGGAAAAAATGTTTTATATGGTTGGGTCCAAAACC
ACAAGTAATAATCATGGACCCTGAGCTTATTAAGGAAGTACTCTCGAAAACCTATTTGTATCAAAAGCCCGGTGGAAATCCATTTGCAGCATTATTG
GTACAAGGACTAGCAACCTATGAAGAAGATAAATGGGCCAAACATAGAAAAATTATCAATCCCGCTTTCCATCTAGAGAAGCTTAAGCATATGCTC
CCAGCTTTTCACTTGAGCTGTACTGAAATGCTGAGCAAATGGGAAGATGCTGTTCCACTTGGCAGCTCTCGTGAGATAGATGTATGGCCTCACCTTC
AGCAATTAACTTGTGATGTAATCTCTCGGACAGCTTTTGGAAGTAGTTATGAAGAAGGTAGAAAGATTTTTGAACTTCAAACGGAACAAGCTCAGA
ATTTTATCGACGCTGTACGTGAAGTTTATATCCCAGGGCGGAGATTTTTGCCAACAAAGAGGAACAGAAGAATGAAGGAAATAAAAAACGAAGTT
CGGACCTCAGTTAAAGGTATTATCGATAAAAGAATGAAGGCAATGAAAGCAGGGAATGCCGATAATAATGAGGATCTGTTAGGCATATTGCTTGA
ATCAAATTTCAAAGAAATTGAACAGCACGGTAACAAGGATTTTGGAATGAGCATCGAAGAAGTCATCGAAGAATGCAAGTTATTCTATTTTGCTGG
CCAAGAAACTACATCAGTCTTACTCGTGTGGACTCTGGTGTTGTTGAGTCGGTATCAAGATTGGCAGACACAGGCCAGAGAAGAAGTCTTGCAAGT
GTTTGAGAGTCGGAAACCAGATTTTGATGGATTAAATCATCTAAAAGTTGTAACAATGATCTTGTACGAGTCATTACGGCTATATCCCCCATTGATA
ACACTTAATCGACAGGTTAACGAAGACATAGTATTAGGAGAATTGTGTCTACCAGCTGGTGTACTAGTCTCATTGCCAATGATCTTATTGCATCATG
ATAAAGATATATGGGGTGAAGATGCAAACGAGTTCAATCCAGAGAGATTTAGAGAAGGAATATCAAGTGCAACAAAAGGTAAAGTTACATATTTT
CCATTTTCATGGGGTCCAAGAATATGCATTGGACAAAATTTTGCCATGTTAGAAGCAAAGATGGCTTTGTGTATGATCCTACAAAGCTTCTCTTTTGA
ATTGTCTCCATCTTACACACATGCTCCTAAATCCTTAGTAACCATGCAGCCTCAGTATGGTGCTCCACTTATTTTGCACAAACTTTAGTTTTGTAATGT
AATGTAACAAGAATTAATATAAAGACTCAAATAAGGGG

SSR2 (Solyc02g069940)

>SL2.50ch02:39361119..39357631
GGGCGTTTGATTGGTAGGCTAAATGGTGGGCTAAAGATATAAATCACACTTACAAAATGATTTTGGATTTTTTTATATTCATTTCATCATTTAAATAT
ATATAATTCATATAAGACGGAGAAATATTGAAAATATTCATAAATTTAGCAAGTTTTTAAATTATGTTATAAGATTCAAGATGTACTATTTAAATTAT
AATTAATAAAGTAAAAATATTATTTTTAATAATTTTTACATATAATTTAAAAGTGTATTTAATACTTAGCTTTTTACGATTTTATCGATGTCCTCTGCCT
ACCAACCAAATAACATGTTTTTCCGTAAATACCCCTATTGTTGTAAGTTCTCTATAAAGCACACTATCATCTTTAACTAAACCATCTCTCATTTTGGGG
AGTAAACTCTGCAATCCAATTATTAGGTACTCATTTTTCATCTCTTCTCAATTTTTTCTCACTATGATTATTATTTTGTTAAATTTAAGCTAATTTTATAG
CTATTTATGCATGAAAAATCATAATTAGTGTTAAGAATTCGGTAGTTAATTATGCTTTTCTTTTTGTACAATGCATGACATAGTTAGAGTAGAACAAA
TTATTTTGTTGCTCATGAGTCATGTTTCACGCATGCTGAAGTTTTTGGGTTTAATTTTTTTTTTGAAGTTAGTTTTATAAACCAATGTGTTGAAAATAA
TTTTCTGTAAAGAACAAATTATATTGTTGCGGAATCAGTTGATAATTTTGAAGTGAAAGAGCATAATCTTATTTTTTGGTAATGCTCCAATAGAAGCA
TATACTAGTAATTTTGTTAGTTTTGCATATCAAAGAAAAATGTTATTAGTTGAAAAATTATGTTGTATATATAGTCAAATTATAAATATTTAAAGCTTG
GATATTTTTGACATAAGTTAGGTTTGAACTTGCTCTGTTTTCGTTTACTACTGTTCTTCCCTAAATTTGAATAAATTATTGAGAAAGCGGAATGGATTA
TTTCTCTTGAGTTTTTTTTTTTTTCTGAATATTTCTATGTTTTTTTTTGTGAATATGGAGGTAAATTTGTCAAAAAGTTTAGATGATTTTTGGAAGACAAC
TTAGATTAGTGATGTTTAACGATTAATTAGTATAAGTTGAGTGACCATTCAGATATTCAACTCCATATTTAGTCTATTTTACCTTTACATCAGTTGATT
TTACAATGATAATTAGTAACAAGAAAATGGCCATGGTGCATAAATTTCAACTTTTAGTTTCAAGATTGTACTGAACTTGGCGTTTTTCGCGGGATGC
TGTTGTTGTTAATCTGACTGTCAATAGCATTATGTTTTTGGGAGAGGTTTCATATTGATGCTATTTGTGCCTTAAAGTTTGTTAAGTAGTGCTCAATT
GGTATGTGATTGATGATAAGTAGTACATTTCTTGGCTGTGGTTCTTAACTTGGATTCGTCGTTGTAACAGTTGTTTGTCCTCAAGTTTTCAAGATGTC
GGATGCTAAGGCCCCCGTGGCCACTGCTTACCCTAAGAGGAAGATCCAGTTGGTGGACTTCCTTCTTTCGTTCCGATGGATCATTGTCATCTTTTTTG
TCCTTCCATTCTCGTTCTTGTATTACTTCTCGATATATCTAGGGGATGTGAAATCCGAGAGGAAATCTTACAAGCAACGCCAGATGGAACACGATGA
GAATGTTAAAGAGGTTGTGAAGCGTCTTGGCCAGAGGAATGCAGAAAAGGATGGTCTTGTATGCACAGCCAGACCTCCTTGGGTGGTTGTTGGAA
TGAGAAATGTCGACTATAAACGTGCTCGTCATTTTGAAGTTGATCTTTCAAAGTTTAGAAATATACTTGATATTGACACGGAGCGGATGGTTGCTAA
AGTTGAGCCTCTAGTCAATATGGGCCAAATGTCAAGGGTCACTATCCCAATGAATCTTTCCCTTGCAGTTCTCGCTGAGCTCGATGATCTTACCGTTG
GTGGTTTGATCAATGGGTTCGGGGTTGAAGGAAGTTCTCACATATTTGGGTTGTTCTCTGACACTGTTGTAGCACTTGAGGTTGTTCTAGCTGATGG
AAAGGTTGTTAGAGCTACAAAGGACAACGAATATTCTGATCTTTTCTACGCTATTCCGTGGTCTCAAGGAACATTGGGGCTTCTTGTTTCAGCTGAA
ATCAAGCTTATACCAGTTGATCAATACGTGAAACTTACCTACAAACCTGTAAGGGGTAATCTTAAAGAGCTTGCTCAGGCTTACGCGGATTCTTTTG
CACCTAAAGATGGAGATCAGGACAATCCTTCTAAAGTTCCTGAGATGGTAGAAGGCATGATTTATGGTCCAACCGAAGGGGTTATGATGACCGGT
ATGTATGCTTCGAGGAATGAAGCCAAACGAAGGGGTAATGTAATCAACAATTACGGTTGGTGGTTCAAACCATGGTTTTACCAACACGCTCAAACC
GCACTAAAAAGAGGGGAATTTGTTGAGTACATTCCAACTAGGGACTACTACCACAGACACACGAGATCGTTGTATTGGGAAGGTAAACTAATTCTT
CCATTCGGTGATCAGTTCTGGTTTAGGTTCCTCTTAGGATGGCTCATGCCACCAAAGATTGCTCTGCTCAAAGCCACACAAAGTGAGGCTATTAGAA
ACTATTACCATGACCATCATGTCATTCAAGATCTCCTTGTTCCTCTTTACAAGGTCGGTGATTGTCTCGAGTGGGTTCATCGCGAGATGGAGGTATAT
CCAATTTGGCTGTGCCCACACAGAATTTACAAGCTGCCAGTGAGACCAATGATCTACCCTGAACCAGGATTCGAGAAACACAAAAGGCAGGGTGA
CACCGAATATGCACAAATGTATACTGATGTTGGTGTGTACTATGTTCCGGGAGCAGTCCTGAGGGGTGAGCCGTTTGATGGTTCAGAGAAATGCCG
ACAACTTGAGCTTTGGTTGATAGAGAACCATGGATTTCAGGCTCAATACGCGGTGACTGAGCTGACAGAGAAGAATTTCTGGAGGATGTTTGATAA
CGGTCTGTACGAGCAATGCAGAAGGAAGTACAAAGCTATCGGAACGTTCATGAGTGTGTACTATAAATCGAAGAAAGGAAGGAAGACAGAGAAG
GAAGTGCAGGAAGCTGAGCAAGAGAAAGCTGAACAAGAGACTCCTGAATTGGTTTCTAGTATGAATTTTCGCCTTTTAAATAAGCAGCTACATTGA

ACAAGTATTTTACTAGTAGTAAGGTCTGCTTGCCTGTTCCAAATTCAAAAAATGTTTCAAATAATAATGGCTAGTGCAGTTCAAAACTCTCAAGTTTC
ATTTCT

HMGR1 (Solyc03g032010)

>SL2.50ch03:4510928..4506920
CCAATCTACCCAGCAGAGTCGCCATTTCTGTTTTATCGAAAAATATTATTTGTTTTGAAAATTTTTTACTTTTAGAGTCGCCACTTAATTATTGAGAAA
AATCAAGAAAAATAACGTTTCTAAAAGACTTATACAGATAAAATCATTTGAAAACATTTTTTAGGGGAGTCCTGGATTCACATTAATATTTTAGGAA
TGTGTTAGGCCCCTAAAATATCCGCTAACTTGCGGTTATCGATAACTAACTTAACTTGGCTAAAATTTAAAATTGTGATCAGATATTTCTTGGAGTTG
TTTGAAAATGAATGTCAAAAATCATTAAGATTGTTTTGAAAGTGAAAAATTATTTTTCTAAATGAGTTTTAATTTGTAAAATCTTTTAAGCCATTTACT
TTATTTGATCGAATCGTTAAAACTAAAGTGGCGTTTTGTGGGGATACGGATTACTCTAACTTTAAACTAGCGACAAACAAAACAACAAATAATTGAA
AAACATATAAAGAGGGAGAGAGATTTGGGTCCAAATCTTGATTTTCTGTTCGCCTTGATCAGAATTTTGGACCCACCTATTTTGGGTTTTTGACCCAT
TTGACTTTCTTTCGTACCTGTGCTAGACTAAATGATAATAAAAAAATAGAGAATATAGAAAAGCAACAAGGGCTTATGCCCAAAATAAATACAATGC
GATAATTTAAAAATAAACTTGGGTTAATTGCCCCAAGTTTTTCAACTCTTCCAACTTGTTTTGAATTCTTCAAATTCTAAACATTTTGACATCCATTCGT
AGGTTTGGGTGACAGATTGACTTGATAAGGAATTTCGAGCCTTGATAGTTCTCTCTCGAAATGCAGAGGGAAGGCGAGAGTTCATAGCGAACTCG
AATGATATCACATATACAATAAAAGAAAAGAGAGAATTAACAAAGAAGCCATAAATTCAACGTCATGCAATTAATTGAAAGTATAATAACATATGT
AGGTAGTGAAGGTGAATGTAAATATAGAAGGAATCATCACTAAGGTTACAATTGAAAGTAATTAGCTTAAAAGTAGTTAAATATAAGTAAAGATCA
TCGGTTGTATAAAAAAAGACATTCTGTAAACTAATAAAGCTATAAATAACCTTAACGATAATAAACATTAAGCAGTAAAATGAAGAAGAAAAAGAA
AAAAAATTTACTAATTTGTCAGAATGAGGTAACGCCGATGTGAGAATTATTATAGATAAATAGATTCAATATATGATAAATCACAAACAAAACTAAA
AGACCTAATGAACTAAATTGTTGACGCCAAAATTGATAGGCAAACGTAATTTATGAAAAAAATATTTTTTTTTACCAATAGACAAAATTATTTTTTTT
CTAAAAAAAAGTAAAAATTTTAATTTAAAGTGTGGCCATAAAAGTCAAGAACTCCAGCCGATGTTTTTTGGGCTATAAATGCATTTCCGATACAAC
AGCTTCAACACTCACAATACTTCCTCACATTTCTCCATTTCTTCTCTGTAAATTCCGGCGACCGGAAAATTTTCACGATGGACGTTCGCCGGCGAGCT
GTAAAACCCCTTGACGCTTCCAAACACATTTCCTCCGGAGAACCTCTCAGACCCCATAATCAAGATTCCTCTGTTAAAGCTTCAGATGCTCTTCCATTA
CCGTTGTATCTTACAAATGGTTTGTTCTTCACCATGTTTTTCTCTGTCATGTATTTTCTTCTACACAGATGGCGTGAGAAGATCCGTAATGGAATTCCA
CTCCACGTGCTCAACTTTTCTGAATTAGTTGCTATCTTTTCGTTGATCGCTTCAGTTATTTATCTGTTGGGGTTCTTTGGTATTGGGTTTGTTCAATCTT
TCGTTTCTAAAGGAAATAATGATTCTTGGGACGTTGAAGATGAAACCCCAGAACAATTTATTGATACAACTGTTACATCACCACCTGTTCGACGAAA
TATCCCAATGAAATCTGTACCTGTTGATGAAAAAGATGCTCAGATAATCACACCATTTTCGTTAGAGGACGATGAGGTGATTATCAAATCGGTGGTG
GAAGGGAGAATACCATCATATTCATTGGAATCTAAGTTGGGTGACTGTAAAAGAGCTGCTTTTATTCGAAAAGAGGCGTTACAGAGGAGTTCAGG
GAAGTCATTGGATGGGTTACCATTAGATGGATTTGATTATGAATCAATTCTTGGACAGTGTTGTGAGATGCCAATTGGGTATATTCAAATACCAGTG
GGAATAGCTGGACCTTTGCTGCTTAATGGGAATGAGTTTTCTGTGCCAATGGCAACCACAGAAGGATGTTTAGTTGCTAGTACTAACAGGGGTTGT
AAAGCCATTTATGTTTCCGGTGGCGCTACCAGTGTTTTGTTTAGAGATGGGATGACTAGAGCTCCTGTAGTTAGGTTCGGCAGCGCAAAGAGAGCT
GCAGAGTTGAAGTTCTTCGTCGAGGATACAATGAATTTCGAGACTCTATCTGTTGTATTCAATAAATCAAGCAGATTTGCCAGATTACAGAACATTC
AATGTGCAATAGCTGGAAAGAATCTATACATGAGGTTTAGCTGTAGCACTGGTGACGCGATGGGAATGAACATGGTTTCCAAAGGTGTACAAAAC
GTTCTTGATTACCTTCAGAATGAGTACCCCGACATGGACATCATTGGCATATCTGGGAACTATTGCTCGGACAAGAAACCAGCAGCAGTTAATTGG
ATTGAGGGGAGAGGAAAGTCGGTAGTTTGTGAGGCAATCATCAAGGAAGAGGTGGTGAAGAAAGTTCTGAAAACTGAGGTTGCTGCTCTAGTTG
AGCTGAACATGCTTAAAAACCTCACTGGCTCTGCCATGGCTGGTGCACTTGGTGGCTTCAACGCCCATGCCAGCAATATTGTCTCAGCTGTATATTT
AGCCACTGGCCAAGACCCGGCTCAAAATATTGAAAGCTCTCACTGCATCACTATGATGGAGGCTGTAAATGATGGCAAGGACCTCCATATATCCGT
CACCATGCCTTCAA

Supplementary Figure 4. G-boxes and GC-rich motifs found in SGA-related genes. The presence of G-boxes (blue), GC-rich motifs (green) and the 5’UTR (pink) present in sense or antisense in the promoter constructs used in the transactivation assays, and the coding sequence (red) are highlighted.

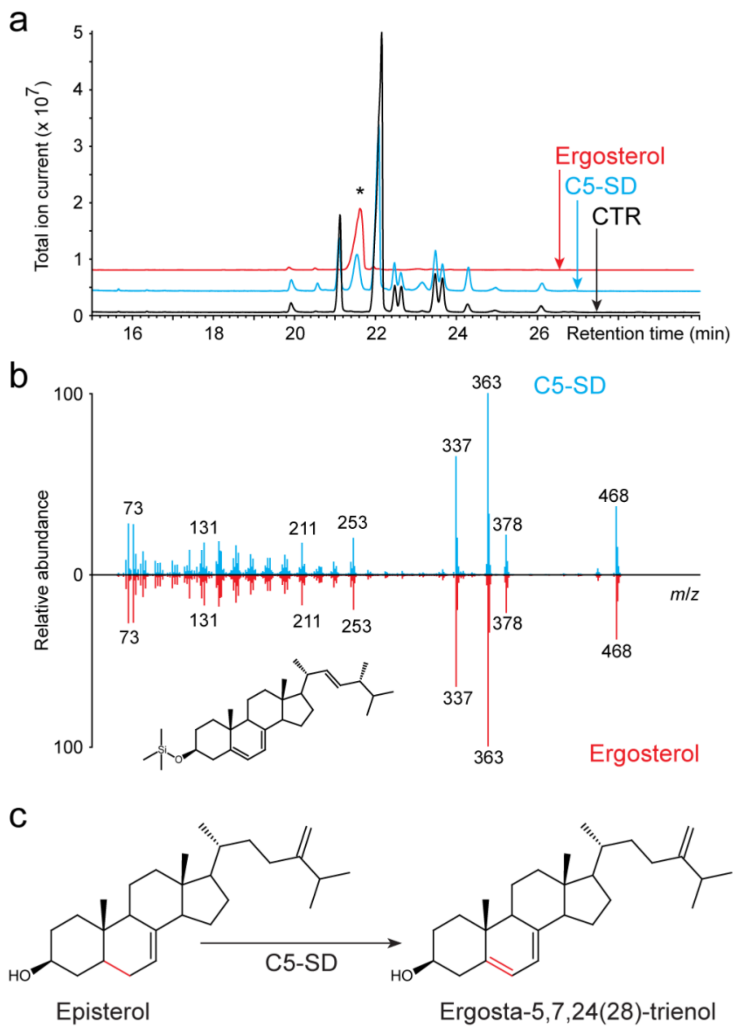

Supplementary Figure 5. *C5-SD* complements a *Saccharomyces cerevisiae erg3* null strain.

(a) Total ion current GC-MS chromatograms of extracts of *erg3Δ* control yeast (CTR, black) and *erg3Δ* yeast expressing *C5-SD* (C5-SD, cyan). The GC-MS chromatogram of an authentic ergosterol standard is also shown (Ergosterol, red). The peak corresponding to ergosterol is indicated with an asterisk.

(b) Comparison of the EI-MS spectra of the ergosterol produced in *erg3Δ* yeast expressing *C5-SD* (C5-SD, cyan) and an authentic ergosterol standard. Samples were trimethylsilylated prior to GC-MS analysis.

(c) Sterol C5 desaturase reaction catalyzed by C5-SD in the *S. cerevisiae erg3* null strain.

a

```
St_GAME9            MNIAIDDDEIFSLPSLDELESITHLLYDD--DSDFFETLSPMSLDSTTLLPNNPTPNSLE
St_GAME9-like1      --MYQQKTTTISDSDLSVLENIKFHLVNDSDFSQILSMFDPINISYA-DIINSPNSSYGS
St_GAME9-like2      ----------MNPSDFSLLQSIQHHLLNDSDIPHIFSAIDSNNTPTN-F-----------
St_GAME9-like5      ---MNPVDDEIFSSDFDLLESMKQYLLNDFDFSEFFS---PSN--NV-ELPNSPTSSFGS
St_GAME9-like3      ----MNINTKFSLSDFDFFESVKQHLLNDFDFSEYFS---PMNLSNV-ELPNSPNSSLGT
St_GAME9-like4      ----MNINTEFSLSDFDFLESVKQHLLNDFDFSEYFS---PMNLSNV-ELPNSPNSSLGS
St_GAME9-like6      ------MQGKISLDIEQNFLDTMQYLFNDPDFSQILSEIQTPRQYSTTSD--HQNAIFPI
St_GAME9-like7      --------------MEHYFSENSDI---------IFSEISSVN----NNN--NNNITIDS
                                      : .              :.

St_GAME9            S-----------------P--------VRPEGTKETFVAREHEESAPQDWRRFIGVRRRQ
St_GAME9-like1      STSAAEISWGDMITNIDNPWQRVDKLEHEEEPKEEPIVARGVH--APGDWNRYRGVRRRP
St_GAME9-like2      --TQNNFDYGELTPLI-NPSTTLQANEKFEVEESKTVAASVTH--APQDWKRYRGVRRRP
St_GAME9-like5      VGNTFLVETTPT----------------KDQTWGPVVARDEHHHVPEDDWRRYIGVRRRQ
St_GAME9-like3      SSSIKSK---------------------EEIIKGPMVVAREEN--AWRDWRRYIGVRRRQ
St_GAME9-like4      SPSIESHEKSES---------------EVEIIKGPMVVTREKH--ASGDWRRYIGVRRRQ
St_GAME9-like6      A---------QNFVQDNIP------------ILQEIEENKESTTPLEHRPKKYKGVRRRP
St_GAME9-like7      S---------QILVNHDIP------------SPTKLSSTEERHVDAPPSWRRYRGVRRRP
                                                                      .:: *****

St_GAME9            WGTFSAEIRDPNRRG-ARLWLGTYESPQDAALAYDQAAYKIRGTKARLNFPDLIGSDVPM
St_GAME9-like1      WGKFAAEIRDPDRKG-ARLWLGTYETPEDAALAYDQAAYKIRGSKARLNFPHLIGSNKSE
St_GAME9-like2      WGKFAAEIRDPDKKN-ARLWLGTYETPEDAALAYDQAAFKIRGSKARLNFPHLIGSGVPE
St_GAME9-like5      WGTFAAEIRDPNRKG-ARLWLGTYETPKDAALAYDDAAFKIRGSKARLNFPHLAGSNISK
St_GAME9-like3      WGTFTAEIRDPDRKG-ARLWLGTYETLKDAALAYD------RGSKARVNFPHLIGSNMPK
St_GAME9-like4      WGTFAAEIRDPDRKG-ARLWLGNYETPEDAALAYDQAAFKIRGSKARVNFSHLIGSNMPE
St_GAME9-like6      WGKYAAEIRDPERKG-CRLWLGTYETPEDAALAYDRTAFKLRGSRAVLNFPHLIESNVTE
St_GAME9-like7      WGKFAAEIRDPKRKGNNRVWLGTYETPEDAALAYDRAAYKMRGARALLNFPHLIGSNVGD
                    **.::******.::   *:***.**: :*******      **::* :** .*  *

St_GAME9            PPRVTARRRTRSRSRSPEPSTTSSSSSS--SSSSSSSSSMENGTKKRKIDLINSIAKAKL
St_GAME9-like1      PIRVAPRRRCHPS----ES------------SLVEY-TS----SKKRKL-----------
St_GAME9-like2      PARVNPRRRSHSP----ES------------SYENV-TP----RKLYTI-----------
St_GAME9-like5      PARVKGSRCLPSP----SS------------SSLSSSSTSENGARKRKIDVINSIAKAKL
St_GAME9-like3      PARLTVRHRTRSP----KP------------SAIS-STSSENGTRKRKIDLINSIAKTNF
St_GAME9-like4      PARLIVRHRTRSP----KP------------SAFS-FTSLENGTRKRKIDLINSIAKAKA
St_GAME9-like6      INRVRPRRRPRSPEFSSSSSPPPPRYVGDNNNESNNNNTDRPSSKRRNVELINSLATVNN
St_GAME9-like7      YNRVYPKRRLNSLDENSIST-----------TKTSNNIECSPSPKRRNVELINSLAKAKA
                      *:   :                       .  .         :  .:

St_GAME9            LCGVNLQMLIQM----------------
St_GAME9-like1      ----------------------------
St_GAME9-like2      ----------------------------
St_GAME9-like5      VCHSLIKFTDVGITLSKERSCFSSCLNL
St_GAME9-like3      FCHSYLKGREFSVQILEKYLPQ------
St_GAME9-like4      KANLFVIIRRGVKT--ER----------
St_GAME9-like6      -LDGQNIMERCLTSN----YYFA-----
St_GAME9-like7      SLNSHCIMERFELGN----LA-------
```

b

```
Sl_GAME9-like4      ------MQGKISLDTEQNFLDT-MQHLF-NDSDFSQILSEIQTPRQYST----TPD----
Sl_GAME9            MSIVIDDDEIFSLP-SLDELESITHLLYDDDSDFFETLSPMSLDVTT--LLPNIPTSNS-
Sl_GAME9-like3      ----MNINMKFSLS-DFDFLESVKQHLL-NDFDFFKYFSPMNLNNVE---LPNSAISSLG
Sl_GAME9-like1      --MYQQKSTTISDS-DLSVLENIKFHLV-NDSDFSQILSMFDPINISHADIINSPNSSYG
Sl_GAME9-like2      ----------MNPS-DFSLLQSIQHHLL-NDSDFPNIFSAIDSNNTPTDF----------
                              :.   . . *:.    *  :* ** : :* :.

Sl_GAME9-like4      ---HQNAIAQ-DN---IPILLQ-----EIEENK--ESTTRLSEH---RPKKYKGVRRRPW
Sl_GAME9            ----------------IESP------VTPEETK--EP-SVACEDAPQDWRRFIGVRRRQW
Sl_GAME9-like3      SSLSIESH--------------EKFEYEEEIIKGPNMVVARQKNTPEDWRRYIGVRRRQW
Sl_GAME9-like1      SSTSAAEISWGDMITNIDSPWQCIDKLEHEEAPKEEPLVARGVHAPGDWNRYRGVRRRPW
Sl_GAME9-like2      ---TQNNFDYGELTPLINSS-TTLQANEKSEVEESETVVASVKHAPKDWKRYRGVRRRPW
                                                 .     :       .     .:: ***** *

Sl_GAME9-like4      GKYAAEIRDPERKGCRLWLGTYETPEDAALAYDRTAFRLRGSKAVLNFPHLIETNVTEIN
Sl_GAME9            GTFSAEIRDPNRRGARLWLGTYESPRDAALAYDQAAYKIRGTKVRLNFPDLIGSDVPMPP
Sl_GAME9-like3      GTFTAEIRDPNKKGARLWLGTYETPEDAALAYDQAAFKIRGSRARVNFPHLIGSNMPKPA
Sl_GAME9-like1      GKFAAEIRDPDRKGARLWLGTYGTPEDAALAYDQAAYKIRGSKARLNFPHLIGSDISEPV
Sl_GAME9-like2      GKFAAEIRDPDKKNARLWLGTYETPEDAALAYDQAAFKIRGSKARLNFPHLIGSGVPEPA
                    *.::******::: .******* :*.*******::*:::**::. :***.** : :

Sl_GAME9-like4      RVRPRRRPRSPEFSSSSPPPPPPPPYVDESNNNNTDGSISKRRNVELINSLATVNNLDCQ
Sl_GAME9            RVTARRRTRSRSRSPEPLTTSSSSSSSSSSSSSSSSENGTKKRKIDLINSIAKSK-LLCG
Sl_GAME9-like3      RLKVRHHTSSLKPSS---------------FSSTSLKNGIRKKK----------------
Sl_GAME9-like1      RVAPRKRCHSSQSSL---------------V------EYTSSKKRKL-------------
Sl_GAME9-like2      RVNPRRRSHSPESSC---------------E------NGTPRKLYFI-------------
                    *:  *::  * . *                       .    :

Sl_GAME9-like4      NIMEKYLTSDYYFA
Sl_GAME9            MDLQMLIQM-----
Sl_GAME9-like3      --------------
Sl_GAME9-like1      --------------
Sl_GAME9-like2      --------------
```

Supplementary Figure 6. Sequence alignment of GAME9 and GAME9-like proteins. Full sequences were aligned with Clustal Omega. The serine-rich C-teminal domain is highlighted in green in GAME9 of potato (**a**) and tomato (**b**).

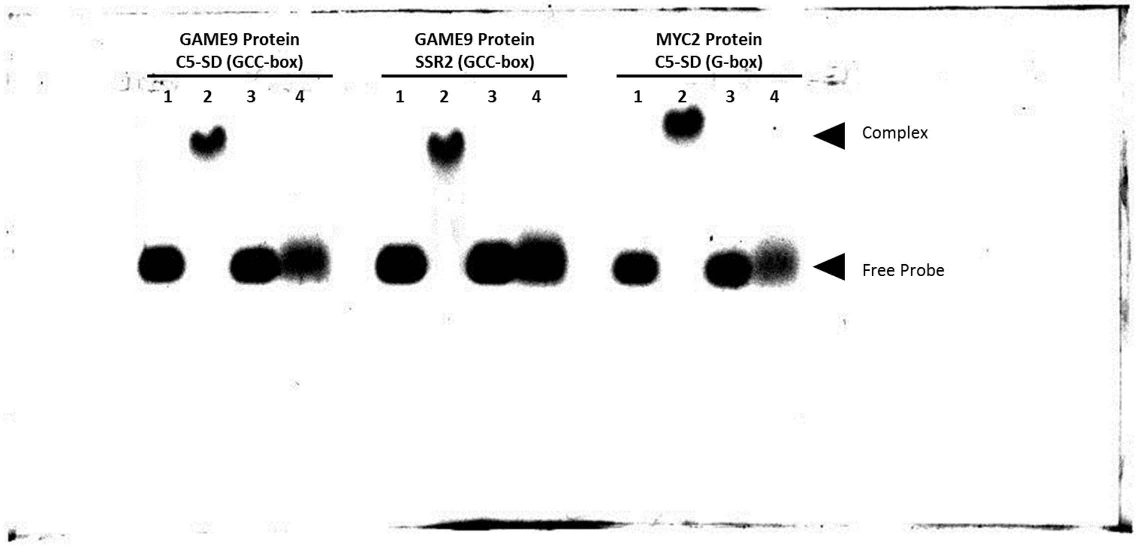

Supplementary Figure 7. *In vitro* binding of the GAME9 and SlMYC2 proteins to the G- and GCC-boxes located in the promoters of the *C5-SD* and *SSR2* genes.

Electrophoretic mobility shift assays (EMSA) were performed with probes containing a GCC- or a G-box were separated on a 1% agarose gel without additional treatment (lane 1) or with 200 ng of the purified protein (lane 2). Controls: a sequence containing a mutated version of the box was loaded (lane 3) and incubated with protein (lane 4).

Supplementary Table 1. Details of homologs co-expressed with *GAME9* in both tomato and potato presented in Fig. 1.

Annotation	Solyc ID (tomato)	Sotub ID (potato)
GAME12 (Aminotransferase-like protein)	Solyc12g006470 [a]	Sotub12g011080 [a]
GAME4 (CYP88D)	Solyc12g006460 [a]	Sotub12g011090 [a]
GAME6 (CYP72A)	Solyc07g043460 [b]	Sotub07g016580 [b]
GAME1 (UDP-galactosyltransferase)	Solyc07g043490 [b]	Sotub07g016600 [b]
GAME11 (2-oxoglutarate-dependent dioxygenase)	Solyc07g043420 [b]	Sotub07g016570 [b]
Cellulose synthase	Solyc07g043390 [b]	Sotub07g016530 [b]
SSR2 (Sterol side chain reductase 2)	Solyc02g069490	Sotub02g015720
Phylloplanin	Solyc01g066680	Sotub01g020750
DNA topoisomerase 2	Solyc01g087500	Sotub01g025950
F-box family protein	Solyc01g104230	Sotub01g041250
Unknown protein	Solyc01g106540	Sotub01g043720
Subtilisin-like serine protease	Solyc02g069630	Sotub02g015850
Ascorbate peroxidase	Solyc02g083630	Sotub02g026940
Male sterility 5 family protein	Solyc02g083640	Sotub02g026950
Laccase	Solyc02g085110	Sotub02g028260
Laccase	Solyc02g085120	Sotub02g028270
Genomic DNA chromosome 5 P1 clone MSI17	Solyc02g086140	Sotub02g029290
Microtubule-associated protein MAP65-1a	Solyc03g007130	Sotub11g029000
Leucine-rich repeat receptor-like protein kinase	Solyc03g083510	Sotub03g020030
E2F transcription factor-like protein	Solyc03g113760	Sotub03g027120
Tubby-like protein 13	Solyc03g117730	Sotub03g031190
Kinesin-like protein	Solyc04g076310	Sotub04g029340
Cyclin A-like protein	Solyc04g078310	Sotub04g031250
Pollen allergen Phl p 11	Solyc05g051870	Sotub05g024500
Glucosyltransferase	Solyc05g053400	Sotub05g026070
Os03g0291800 protein	Solyc06g050920	Sotub06g011200
Cyclin-dependent protein kinase regulator-like protein	Solyc06g065680	Sotub06g024730
Genomic DNA chromosome 5 TAC clone K19B1	Solyc06g070970	Sotub06g026960
Leucine-rich repeat receptor-like protein kinase PEPR2	Solyc06g084420	Sotub06g034920
Dopamine beta-monooxygenase	Solyc07g048050	Sotub07g018160
LRR receptor-like serine/threonine-protein kinase	Solyc08g061560	Sotub08g014090
Pectinesterase	Solyc08g078640	Sotub08g024430
Os04g0690100 protein	Solyc08g079010	Sotub08g024870
Alpha-hydroxynitrile lyase	Solyc09g014970	Sotub09g012700
Cyclin A1	Solyc11g005090	Sotub11g013250
Superoxide dismutase	Solyc11g066390	Sotub11g024220
Genomic DNA chromosome 5 P1 clone MUP24	Solyc11g069420	Sotub11g026580

[a,b]Genes localized in clusters on chromosome 12 and 7, respectively, previously associated with SGA biosynthesis.

Supplementary Table 2. Changes in expression of genes involved in the biosynthesis of SGAs and sterol precursors in potato *GAME9*-Ox leaves determined by RNA-Seq analyses.

Process	Gene	Id	Description	Change
Regulation	*GAME9*	Sotub01g029510	Ethylene responsive transcription factor	↑
SGA biosynthesis (Chr 7)	*GAME2*	Sotub07g016550	UDP-rhamnosyltransferase	↑
	GAME11	Sotub07g016570	2-oxoglutarate-dependent dioxygenase	↑
	GAME6	Sotub07g016580	Cytochrome P450 72A188	↑
	GAME1	Sotub07g016600	UDP-galactosyltransferase	↑
SGA biosynthesis (Chr 12)	*GAME12*	Sotub12g011080	Aminotransferase-like protein	↑
	GAME4	Sotub12g011090	Cytochrome P450 88D	↑
Biosynthesis of precursors	*HMGR*[*a]	Sotub02g025630	3-Hydroxy-3-methylglutaryl CoA reductase 1	No change
	SQS[*a]	Sotub01g047720	Squalene synthase	No change
	CAS1	Sotub04g023080	Cycloartenol synthase	↑
	TTS1[*b]	Sotub12g010880	*β*-amyrin synthase	No change
	SMT1[*a]	Sotub01g025880	Sterol C24-methyltransferase type1	No change
	SSR2[*c]	Sotub02g015720	Sterol side chain reductase 2	↑

Identified by *[a]Ginzberg *et al.*[35], *[b]Wang *et al.*[39] and *[c]Sawai *et al.*[19]

Genes are grouped according to their association with SGA biosynthesis or synthesis of precursors. Change is indicated when increase (↑) in gene expression was found as compared to wild-type potato plants.

Supplementary Table 3. Changes in expression of genes involved in the biosynthesis of SGAs and precursors in tomato *GAME9*-RNAi and *GAME9*-Ox leaves as determined by RNA-Seq analyses.

Process	Gene	Id	Description	RNAi	Ox
Regulation	*GAME9*	Solyc01g090340	Ethylene responsive transcription factor	↓	↑
SGA biosynthesis (Chr 7)	*GAME2*	Solyc07g043410	UDP-xylosyltransferase	No change	No change
	GAME11	Solyc07g043420	2-oxoglutarate-dependent dioxygenase	↓	↑
	GAME6	Solyc07g043460	Cytochrome P450 72A188	↓	↑
	GAME17	Solyc07g043480	UDP-glucosyltransferase	↓	↑
	GAME1	Solyc07g043490	UDP-galactosyltransferase	↓	↑
	GAME18	Solyc07g043500	UDP-glucosyltransferase	↓	↑
SGA biosynthesis (Chr 12)	*GAME12*	Solyc12g006470	Aminotransferase-like protein	↓	↑
Biosynthesis of precursors	*SQS*[*a]	Solyc01g110290	Squalene synthase	No change	No change
	TTS1[*b]	Solyc12g006530	*β*-amyrin synthase	No change	No change
	TTS2[*b]	Solyc12g006520	*β*-amyrin synthase	No change	No change
	SSR2[*c]	Solyc02g069490	Sterol side chain reductase 2	↓	↑

Identified by *[a]Ginzberg *et al*.[35], *[b]Wang *et al*.[39] and *[c]Sawai *et al*.[19]

Genes are grouped according to their association with SGA biosynthesis or synthesis of precursors. Change is indicated when increase (↑) or decrease (↓) in gene expression was found in comparison to wild-type tomato plants.

Supplementary Table 4. A set of 27 genes (including GAME9) found in common between the down- and upregulated genes in the *GAME9*-RNAi and *GAME9*-Ox tomato lines, respectively.

Solyc ID	Annotation
Solyc01g090340	Ethylene responsive transcription factor (GAME9)
Solyc07g043490[a]	UDP-galactosyltransferase (GAME1)[b]
Solyc07g043480[a]	UDP-glucosyltransferase (GAME17)[c]
Solyc07g043420[a]	2-oxoglutarate-dependent dioxygenase (GAME11)[b]
Solyc07g043460[a]	CYP72A (GAME6)[b]
Solyc07g043500[a]	UDP-glucosyltransferase (GAME18)[c]
Solyc02g069490	Sterol side chain reductase 2 (SSR2)[b]
Solyc02g086180	Δ(7)-sterol-C5(6)-desaturase (C5-SD)[c]
Solyc06g005750	Methylsterol monooxygenase 2-2-like (SMO1)[c]
Solyc01g073640	3-*β*-hydroxysteroid dehydrogenase (OXR)[c]
Solyc10g008410	E3 ubiquitin-protein ligase RMA1H1-like (UBL)[b]
Solyc07g043390[a]	Cellulose synthase[b]
Solyc09g018660	Unknown protein
Solyc03g051690	Alcohol dehydrogenase (fragment)
Solyc10g074660	Unknown protein
Solyc12g032960	Photosystem I P700 chlorophyll a apoprotein
Solyc09g074480	Unknown protein
Solyc09g009070	Repressor of silencing 1
Solyc01g017320	Photosystem I P700 chlorophyll a apoprotein A1
Solyc06g009950	Photosystem I P700 chlorophyll a apoprotein A2
Solyc03g078030	Unknown protein
Solyc02g036320	Photosystem I P700 chlorophyll a apoprotein
Solyc05g007830	Expansin 2
Solyc04g007480	Unknown Protein
Solyc06g074590	Ycf2
Solyc01g007390	Unknown protein
Solyc12g056290	Unknown protein

[a]Genes localized in the chromosome 7 SGAs gene cluster.

[b]Genes co-expressed with *GAME9* in potato and tomato.

[c]Genes co-expressed with *GAME9* in tomato.

Supplementary Table 5. Details of promoters tested in tobacco protoplast transfection assays for transactivation by GAME9.

Gene Symbol	Annotation	Solyc ID	Size cloned
GAME1	UDP-galactosyltransferase	Solyc07g043490	~2600 bp
GAME2	UDP-xylosyltransferase	Solyc07g043410	~2300 bp
GAME4	CYP88D	Solyc12g006460	~2600 bp
GAME6	CYP72A	Solyc07g043460	~2600 bp
GAME7	CYP72A	Solyc07g062520	~2700 bp
GAME17	UDP-glucosyltransferase	Solyc07g043480	~1900 bp
GAME18	UDP-glucosyltransferase	Soly07g043500	~1550 bp
HMGR1	HMG CoA reductase 1	Solyc03g032010	~1500 bp
HMGR2	HMG CoA reductase 2	Solyc03g032020	~1500 bp
SQMO	Squalene monooxygenase	Solyc04g077440	~1200 bp
SMO1	Methylsterol monooxygenase 2-2-like	Solyc06g005750	~2250 bp
C5-SD	Δ(7)-sterol-C5(6)-desaturase	Solyc02g086180	~1600 bp

For each gene, a region spanning ~1200-2700 bp was cloned and used for transactivation in tobacco protoplasts.

Supplementary Table 6. Oligonucleotides used in this study.

	Name	Sequence	Use
C5-SD promoter	C5SD_Fw3	GGGGACAAGTTTGTACAAAAAAGCAGGCTTAAATCTTCGCAAAATAATAGATA	Deletion C5-SD d3
	C5SD_Rv1	GGGGACCACTTTGTACAAGAAAGCTGGGTACTCCGACGGTATACTAGGTA	Deletion C5-SD d3
	C5SD_Fw4	GGGGACAAGTTTGTACAAAAAAGCAGGCTTAGAAACATGACAAGGTGAATTC	Deletion C5-SD d4
	C5SD_Rv1	GGGGACCACTTTGTACAAGAAAGCTGGGTACTCCGACGGTATACTAGGTA	Deletion C5-SD d4
	C5SD_Fw6	GGGGACAAGTTTGTACAAAAAAGCAGGCTTAAATCTTTTGCCAGTTTAGCTC	Deletion C5-SD d6
	C5SD_Rv1	GGGGACCACTTTGTACAAGAAAGCTGGGTACTCCGACGGTATACTAGGTA	Deletion C5-SD d6
	C5SD_Fw7	GGGGACAAGTTTGTACAAAAAAGCAGGCTTAGGAGTAGTAGTCCAAAATGG	Deletion C5-SD d7
	C5SD_Rv1	GGGGACCACTTTGTACAAGAAAGCTGGGTACTCCGACGGTATACTAGGTA	Deletion C5-SD d7
	C5SD_Fw5	GGGGACAAGTTTGTACAAAAAAGCAGGCTTACACATGCATATATTGTGTTACC	Deletion C5-SD d5
	C5SD_Rv1	GGGGACCACTTTGTACAAGAAAGCTGGGTACTCCGACGGTATACTAGGTA	Deletion C5-SD d5
	C5SD_Fw4	GGGGACAAGTTTGTACAAAAAAGCAGGCTTAGAAACATGACAAGGTGAATTC	Deletion C5-SD d8
	C5SD_Rv2	GGGGACCACTTTGTACAAGAAAGCTGGGTATAGTGCGGTAACACAATATATG	Deletion C5-SD d8
	C5SD_Fw6	GGGGACAAGTTTGTACAAAAAAGCAGGCTTAAATCTTTTGCCAGTTTAGCTC	Deletion C5-SD d9
	C5SD_Rv2	GGGGACCACTTTGTACAAGAAAGCTGGGTATAGTGCGGTAACACAATATATG	Deletion C5-SD d9
	Gbox_Fw	CAGTTTAGCTCACCACACAAatgtgaGGAGTAGTAGTCCAAAATGG	Mutation mG-box
	Gbox_Rv	CCATTTTGGACTACTACTCCtcacatTTGTGTGGTGAGCTAAACTG	Mutation mG-box
	GCC_Fw	GAATCAATTTTCATTAAATACgattacagtcACATGCATATATTGTGTTAC	Mutation mGCC-box
	GCC_Rv	GTAACACAATATATGCATGTgactgtaatcGTATTTAATGAAAATTGATTC	Mutation mGCC-box
Potato	NAC-Fw	ATATAGAGCTGGTGATGACT	qRT-PCR
	NAC-Rv	TCCATGATAGCAGAGACTA	qRT-PCR
	GAME9-Fw	AAGCCGCTTACAAGATTCGG	qRT-PCR (also used in tomato)
	GAME9-Rv	ACGACGCCTAGCCGTTACTC	qRT-PCR (also used in tomato)
	CAS-Fw	AATCATGACGGTCACTGGGCT	qRT-PCR
	CAS-Rv	AATACTGCATTGAGTGCCCCC	qRT-PCR
	SMT1-Fw	GCTTTGCTGTGTATGAGTGG	qRT-PCR
	SMT1-Rv	TTTTGTGTCGATCGAATCTC	qRT-PCR
	SSR2-Fw	CCACCGTTCACCCTAGGAGG	qRT-PCR
	SSR2-Rv	ATACAAGAACGAGAATGGAAGGACA	qRT-PCR
	GAME11-Fw	TGGCGGACCTTCTTTCAAACT	qRT-PCR
	GAME11-Rv	CACAATTTCAACTGGATCCGATG	qRT-PCR
	GAME6-Fw	TTTGGCCGTATGTTTGCCTT	qRT-PCR
	GAME6-Rv	TTCCTTGCTGCCGCAGTT	qRT-PCR

	GAME4-Fw	GGGACTCAAGGCTCGAAAAGTACT	qRT-PCR
	GAME4-Rv	TGTTTGCCCTTGGCATTGAT	qRT-PCR
	GAME12-Fw	GGAATGGCCAAGACTACTAATGGA	qRT-PCR
	GAME12-Rv	GCTCTATCTATAACTAAAGGTCCCATA	qRT-PCR
	GAME1-Fw	TGGGTCCCACAGCTTACGATC	qRT-PCR
	GAME1-Rv	GGCACGCCAAAAGTGATGG	qRT-PCR
	GAME2-Fw	GAAACTGCAATGCCGCATGT	qRT-PCR
	GAME2-Rv	TTGAGGCCATGGAGGGC	qRT-PCR
	C5-SD Fw	TCATCTCCGGTTTCCTCTGGT	qRT-PCR
	C5-SD Rv	TTGCAAGAGCATTGCTTCCTTT	qRT-PCR
	GAME9-Ox-Fw	GGGGACAAGTTTGTACAAAAAAGCAGGCTATGAATATTGCAATTGATGATGATGA	Cloning GAME9 for Ox
	GAME9-Ox-Rv	GGGGACCACTTTGTACAAGAAAGCTGGGTTCATTTGTATCAACATTTGTAAATTCACAC	Cloning GAME9 for Ox
	GAME9-RNAi-Fw	AAAAAGCGGCCGCATGAGTATTGTAATTGATGATGATGAAATC	Cloning GAME9 for RNAi (also used in tomato)
	GAME9-RNAi-Rv	AAAAAGGCGCGCCCACACGCCACAGATGGTTCTT	Cloning GAME9 for RNAi (also used in tomato)
Tomato	TIP41-Fw	AGATGAACTGGCAGATAATGG	qRT-PCR
	TIP41-Rv	CATCAACCCTAAGCCAGAAA	qRT-PCR
	HMGR-Fw	CTGACGCGCTTCCACTCC	qRT-PCR
	HMGR-Rv	GATCTTCTCACGCCACCTTACG	qRT-PCR
	SSR2-Fw	GGCCAAATGTCAAGGGTCACT	qRT-PCR
	SSR2-Rv	ACCCCGAACCCATTGATCA	qRT-PCR
	GAME11-Fw	TGGTCCTGAGAATCCTCCACA	qRT-PCR
	GAME11-Rv	GCTCCAATGAAGCGTGGTACAC	qRT-PCR
	GAME4-Fw	CTTCAATGTGTGGTGATCCAAAA	qRT-PCR
	GAME4-Rv	CCATAATTGTTGGCTTCCCAAA	qRT-PCR
	GAME12-Fw	TATGACTGCCGGTCTCTCCG	qRT-PCR
	GAME12-Rv	GATAGTTCCAATAATGAGGGCAATCA	qRT-PCR
	GAME17-Fw	GCTGCAGGATTCCCTATTCCAC	qRT-PCR
	GAME17-Rv	TACTTAGCATGGTGCTCCAC	qRT-PCR
	C5-SD Fw	TCATCTCCGGTTTCCTCTGGT	qRT-PCR
	C5-SD Rv	TTGCAACAGCATTGCTTGCT	qRT-PCR
	GAME9-Ox-Fw	GGGGACAAGTTTGTACAAAAAAGCAGGCTATGAGTATTGTAATTGATGATGATGAAATC	Cloning GAME9 for Ox
	GAME9-Ox-Rv	GGGGACAAGTTTGTACAAAAAAGCAGGCTATGAGTATTGTAATTGATGATGATGAAATC	Cloning GAME9 for Ox
	C5-SD-Fw	GTGCATGATTTGCACTCAAGGATG	VIGS
	C5-SD-Rv	CCACCATAACATGGTGACAATGTG	VIGS
	C5-SD-Fw	GGGGACAAGTTTGTACAAAAAAGCAGGCTATGGAGGATTACTTGAAGCTATTCG	Yeast expression
	C5-SD-Rv	GGGGACCACTTTGTACAAGAAAGCTGGGTTTACATTTTCTTGGCATCCTCTTC	Yeast expression

The underlined sequences correspond to the gateway sequence. The sequences in lowercase represent the mutated G- or GCC-boxes.

Supplementary Table 7. Probes used for electrophoretic mobility shift assays.

C5-SD (GCC-box)	C5-SD Fw	AATCAATTTTCATTAAATACAGCCTGCCACACATGCATATATTGTGTTA
	C5-SD Rv	TAACACAATATATGCATGTGTGGCAGGCTGTATTTAATGAAAATTGATT
C5-SD (mutated GCC-box) Negative Control	C5-SD NTC Fw	AATCAATTTTCATTAAATACATCTTTTGCCACATGCATATATTGTGTTA
	C5-SD NTC Rv	TAACACAATATATGCATGTGGCAAAAGATGTATTTAATGAAAATTGATT
SSR2 (GCC-box)	SSR2 Fw	GTGGGGGCGTTTGATTGGTAGGCTAAATGGTGGGCTAAAGATATAAATCAC
	SSR2 Rv	GTGATTTATATCTTTAGCCCACCATTTAGCCTACCAATCAAACGCCCCCAC
SSR2 (mutated GCC-box) Negative Control	SSR2 NTC Fw	GTGGGGGCGTTTGATATCTTTTGCAAAATCTTTTGCAAAGATATAAATCAC
	SSR2 NTC Rv	GTGATTTATATCTTTGCAAAAGATTTTGCAAAAGATATCAAACGCCCCCAC
C5-SD (G-box)	G-box Fw	TAGCTCACCACACAACACGTGGGAGTAGTAGTCCAA
	G-box Rv	TTGGACTACTACTCCCACGTGTTGTGTGGTGAGCTA
C5-SD (mutated G-box) Negative Control	G-box NTC Fw	TAGCTCACCACACAAAAAAAAGGAGTAGTAGTCCAA
	G-box NTC Rv	TTGGACTACTACTCCTTTTTTTTGTGTGGTGAGCTA

The underlined sequences correspond to the GCC- and G-boxes.

Supplementary Table 8. Accession numbers for ERF proteins used in the phylogenetic analysis presented in Fig. 2.

Name in Figure 2	Organism	Accession number
Sl_GAME9-like1	*Solanum lycopersicum*	Solyc01g090300
Sl_GAME9-like2	*Solanum lycopersicum*	Solyc01g090310
Sl_GAME9-like3	*Solanum lycopersicum*	Solyc01g090320
Sl_GAME9	*Solanum lycopersicum*	Solyc01g090340
Sl_GAME9-like4	*Solanum lycopersicum*	Solyc01g090370
St_GAME9-like1	*Solanum tuberosum*	PGSC0003DMG400026049
St_GAME9-like2	*Solanum tuberosum*	PGSC0003DMG400041045
St_GAME9-like3	*Solanum tuberosum*	PGSC0003DMG400025991
St_GAME9-like4	*Solanum tuberosum*	PGSC0003DMG400046672
St_GAME9	*Solanum tuberosum*	PGSC0003DMG400025989
St_GAME9-like5	*Solanum tuberosum*	PGSC0003DMG400026048
St_GAME9-like6	*Solanum tuberosum*	PGSC0003DMG400026046
St_GAME9-like7	*Solanum tuberosum*	PGSC0003DMG400040573
Nt_ERF189	*Nicotiana tabacum*	ERF189
Nt_ERF115	*Nicotiana tabacum*	ERF115
Nt_ERF179	*Nicotiana tabacum*	ERF179
Nt_ERF168	*Nicotiana tabacum*	ERF168
Nt_ERF221/ORC1	*Nicotiana tabacum*	ERF221
Nt_ERF104	*Nicotiana tabacum*	ERF104
Nt_ERF17	*Nicotiana tabacum*	ERF17
Cr_ORCA1	*Catharanthus roseus*	AJ238739
Cr_ORCA2	*Catharanthus roseus*	AJ238740
Cr_ORCA3	*Catharanthus roseus*	EU072424
Sl_SHN3	*Solanum lycopersicum*	XP_004240977.1
Sl_SHN1	*Solanum lycopersicum*	XP_004235965.1
At_SHN1	*Arabidopsis thaliana*	At1g15360
At_SHN2	*Arabidopsis thaliana*	At5g11190
At_SHN3	*Arabidopsis thaliana*	At5g25390
Sl_ERF1	*Solanum lycopersicum*	AAL75809
Os_SHN1	*Oryza sativa*	BAD15859
At_ERF1	*Arabidopsis thaliana*	At4g17500
At_DREB1A	*Arabidopsis thaliana*	At4g25480
At_DREB1B	*Arabidopsis thaliana*	At4g25490
At_DREB1C	*Arabidopsis thaliana*	At4g25470
At_DREB2A	*Arabidopsis thaliana*	At5g05410
At_DREB2B	*Arabidopsis thaliana*	At3g11020
Sl_AP2a	*Solanum lycopersicum*	ACD62792
Sl_AP2b	*Solanum lycopersicum*	HQ586952
Sl_AP2c	*Solanum lycopersicum*	HQ586951
Sl_AP2d	*Solanum lycopersicum*	HQ586953

Sl_AP2e	*Solanum lycopersicum*	HQ586954
At_ANT	*Arabidopsis thaliana*	Q38914
At_TOE2	*Arabidopsis thaliana*	Q9LVG2
At_ WRI1	*Arabidopsis thaliana*	AY254038
At_AP2	*Arabidopsis thaliana*	At4g369200
Bn_BBM1	*Brassica napus*	AF317904
Bn_BBM2	*Brassica napus*	AF317905
At_PLT1	*Arabidopsis thaliana*	At3g20840

Sequence data can be found in GenBank/EMBL data libraries, Sol Genomics Network or in the tobacco transcription factors (TOBFAC) database, respectively.
